# Eukaryotic initiation factor 6 regulates mechanical responses in endothelial cells

**DOI:** 10.1083/jcb.202005213

**Published:** 2022-01-13

**Authors:** Adam N. Keen, Luke A. Payne, Vedanta Mehta, Alistair Rice, Lisa J. Simpson, Kar Lai Pang, Armando del Rio Hernandez, John S. Reader, Ellie Tzima

**Affiliations:** 1 Radcliffe Department of Medicine, University of Oxford, Oxford, UK; 2 Wellcome Centre for Human Genetics, University of Oxford, Oxford, UK; 3 Cellular and Molecular Biomechanics Laboratory, Department of Bioengineering, Imperial College London, London, UK

## Abstract

The repertoire of extratranslational functions of components of the protein synthesis apparatus is expanding to include control of key cell signaling networks. However, very little is known about noncanonical functions of members of the protein synthesis machinery in regulating cellular mechanics. We demonstrate that the eukaryotic initiation factor 6 (eIF6) modulates cellular mechanobiology. eIF6-depleted endothelial cells, under basal conditions, exhibit unchanged nascent protein synthesis, polysome profiles, and cytoskeleton protein expression, with minimal effects on ribosomal biogenesis. In contrast, using traction force and atomic force microscopy, we show that loss of eIF6 leads to reduced stiffness and force generation accompanied by cytoskeletal and focal adhesion defects. Mechanistically, we show that eIF6 is required for the correct spatial mechanoactivation of ERK1/2 via stabilization of an eIF6–RACK1–ERK1/2–FAK mechanocomplex, which is necessary for force-induced remodeling. These results reveal an extratranslational function for eIF6 and a novel paradigm for how mechanotransduction, the cellular cytoskeleton, and protein translation constituents are linked.

## Teaser

Keen et al. discover a new role for eIF6, a protein classically known for its role in protein synthesis, in mechanotransduction, and the cellular response to force. These data provide a novel paradigm for how the cytoskeletal and translational machineries are linked.

## Introduction

Cells respond and adapt to a variety of mechanical stresses that regulate cellular signaling and function. Whether externally applied or internally generated, forces are transduced via the cytoskeleton machinery, an intricate fibrous network that provides the structural architecture and governs shape, size, and mechanical properties of the cell (Harris et al., 2016; Pegoraro et al., 2017; Wang et al., 1993). The cytoskeleton is anchored to the base of the cell by large macromolecular complexes with both mechanical and cell signaling components, called focal adhesions (DeMali et al., 2003; Mitra et al., 2005). Focal adhesions constitute well-described sites of mechanosensing; cytoskeletally generated forces lead to stresses in these adhesions because of the opposite forces that arise in the ECM. Focal adhesions are highly dynamic, requiring the correct spatiotemporal activation of signaling cascades, including FAK and extracellular signal-regulated kinase 1/2 (ERK1/2; Fincham et al., 2000; Mitra and Schlaepfer, 2006; Parsons, 2003). Externally applied forces (through the ECM, ion channels, or other mechanoreceptors) also trigger active changes in cytoskeletal structures and cellular force generation. Force application on integrins (Choquet et al., 1997) or cell adhesion molecules, such as platelet endothelial cell adhesion molecule-1 (PECAM-1; Barry et al., 2015; Collins et al., 2012; Collins et al., 2014) or cadherins (Barry et al., 2015; Bays et al., 2017; Muhamed et al., 2016), leads to signaling cascades that ultimately lead to growth of adhesions and reinforcement of the cytoskeleton.

In addition to structural roles, the cytoskeleton modulates many cellular processes by providing a structural/physical platform that influences the activity and/or subcellular localization of signaling proteins and their downstream targets (Bezanilla et al., 2015; Harris et al., 2016; Janmey, 1998). Elaborate and functionally important interactions between components of the cytoskeleton and the protein synthesis apparatus suggest coregulation between these two cellular machineries (Fujimura et al., 2015; Gross and Kinzy, 2005; Horton et al., 2015; Kim and Coulombe, 2010; Liu et al., 2002; Simpson et al., 2020a, 2020b; Smart et al., 2003; Tzima et al., 2003; Willett et al., 2010). However, our understanding of this is rudimentary at best.

Throughout evolution, members of the protein synthesis apparatus have been co-opted to carry out auxiliary extratranslational functions (Diebel et al., 2016; Guo and Schimmel, 2013; Mateyak and Kinzy, 2010; Warner and McIntosh, 2009). An example of this is the highly conserved receptor of activated C kinase 1 (RACK1) protein, which in addition to binding to the small 40S ribosomal subunit to prevent unproductive 80S monosome formation (Gallo and Manfrini, 2015), is also an integrin-binding and cytoskeleton-regulating protein (Liliental and Chang, 1998). The ability of RACK1 to interact with several proteins has supported the model that RACK1 functions as a linker between the cell signaling and translation machineries (Gallo and Manfrini, 2015). A RACK1-interacting protein of interest is the eukaryotic initiation factor 6 (eIF6; Ceci et al., 2003; Gallo and Manfrini, 2015; Grosso et al., 2008). Originally identified as an integrin-binding protein itself (Biffo et al., 1997) and recently identified in proteomic analysis of integrin adhesion complexes (Byron et al., 2015), eIF6 can also bind to the large 60S ribosomal subunit and act as a chaperone in a way analogous to RACK1/2 to regulate formation of an active 80S ribosome capable of protein translation but preventing unproductive ribosomal subunit joining in the absence of mRNA (Ceci et al., 2003; Gandin et al., 2008; Russell and Spremulli, 1979; Valenzuela et al., 1982; Warren, 2018). A number of elegant structural studies have now shown that the ribosome maturation factor Shwachman–Bodian–Diamond syndrome protein, in combination with elongation factor-like GTPase 1, removes eIF6 from the 60S (Warren, 2018; Weis et al., 2015; Wong et al., 2011), allowing 80S formation and, therefore, protein elongation to proceed. In addition, eIF6 has been shown to be important for ribosome biogenesis (Basu et al., 2001; Brina et al., 2015a; Sanvito et al., 1999). Interestingly, perturbations of eIF6 or RACK1 do not have any observable effects on steady-state translation (Gandin et al., 2008; Volta et al., 2013), but they do impair translational upregulation in response to certain stimuli (e.g., insulin; Brina et al., 2015b; Gandin et al., 2008; Miluzio et al., 2016). eIF6 has been linked to a variety of processes, including tumor biology (Miluzio et al., 2015; Sanvito et al., 2000) and regulation of metabolism (Brina et al., 2015b; Miluzio et al., 2016), and importantly, noncanonical roles of eIF6 in wound healing have been reported (Shu et al., 2016; Yang et al., 2015). However, the role of eIF6 in endothelial cells (ECs) and/or mechanotransduction has not been investigated. Here, we used a loss-of-function approach to determine if there is a dual role for eIF6 in protein synthesis and mechanosignaling in ECs. We show that although depletion of eIF6 does not affect steady-state nascent protein synthesis and only has minimal effects on ribosomal biogenesis in unstimulated cells, eIF6 regulates cell mechanics and the endothelial response to force via the dynamic activation of mechanotransduction pathways to ultimately regulate endothelial mechanics.

## Results

### Effects of eIF6 depletion on protein synthesis and ribosomal biogenesis in ECs

**Figure 1.**
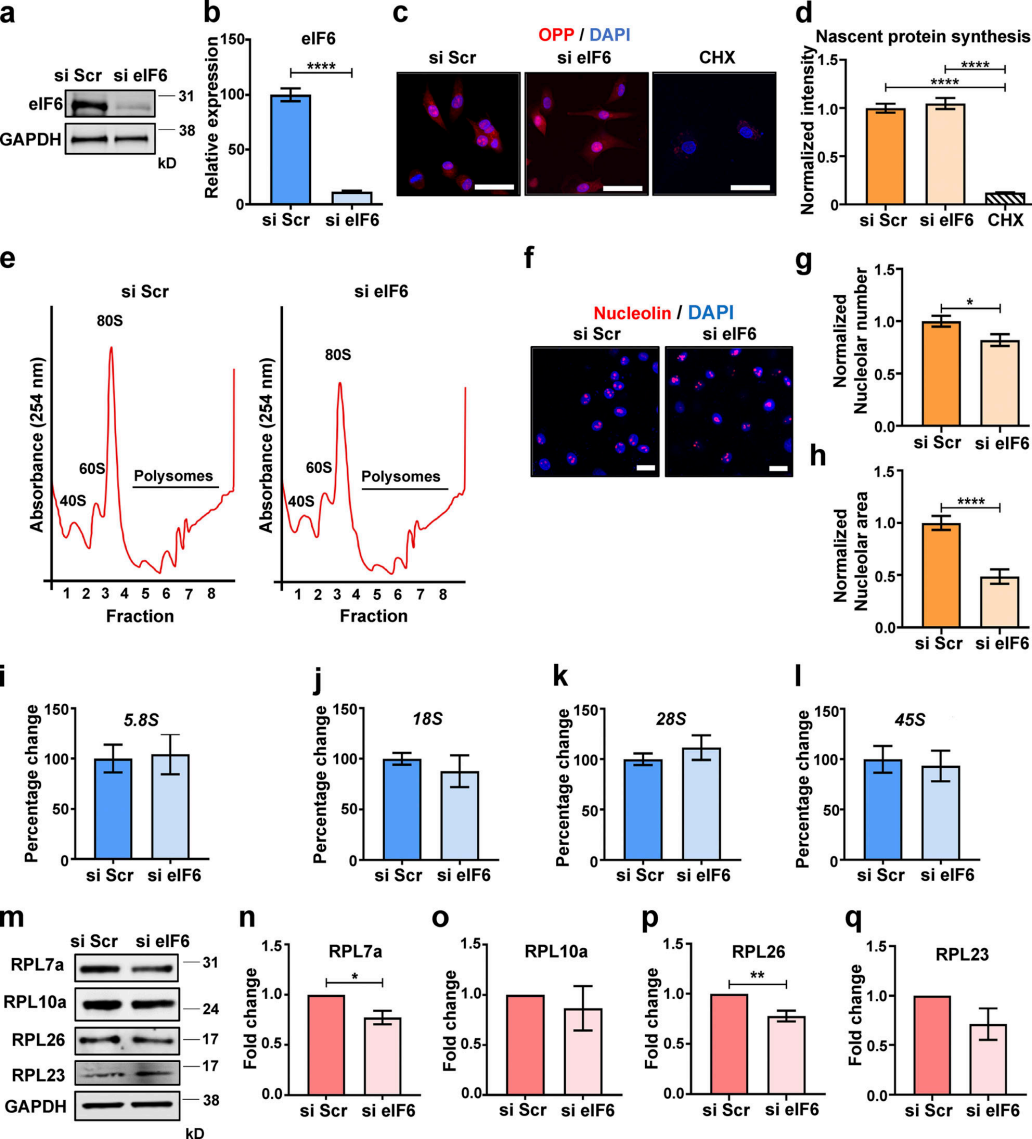
**Depletion of endogenous eIF6 does not affect basal levels of protein synthesis or ribosome biogenesis. (a and b)** Representative Western blot of si Scr– or si eIF6–transfected ECs and quantification of knockdown efficiency (*n* = 4). **(c)** Representative fluorescent micrographs of si Scr– or si eIF6–transfected ECs, or cycloheximide (CHX)-treated ECs following incorporation of OPP to label nascent proteins (red) using a Click-iT assay and costaining of cell nuclei (DAPI; blue). Scale bars = 20 μm. **(d)** Quantification of cell fluorescence following OPP incorporation Click-iT assay (*n* > 30 cells across three separate experiments). **(e)** Representative polysome profiles from si Scr and si eIF6 A431 cells after sucrose gradient fractionation, showing the small ribosomal subunit (40S), the large ribosomal subunit (60S), and the monoribosome (80S; *n* = 3). **(f)** Representative immunofluorescent micrographs of si Scr and si eIF6 ECs showing nucleolin (red) and cell nuclei (DAPI; blue). Scale bars = 20 μm. **(g and h)** Quantification of nucleolar frequency per cell (g) and nucleolar area (h; *n* > 30 and *n* > 60, respectively, across three separate experiments). **(i–l)** Quantification of pre-rRNA by qPCR in si Scr– and si eIF6–transfected ECs relative to GAPDH (*n* > 3): *5.8S* rRNA (i), *18S* rRNA (j), *28S* rRNA (k), and *45S* rRNA (l). **(m–q)** Quantification of ribosomal protein expression in si Scr– and si eIF6–transfected ECs, representative Western blots (m) and band intensity quantification of RPL7a (n), RPL10a (o), RPL26 (p), and RPL23 (q; *n* > 3). Values in b, d, g–l, and n–q are mean ± SEM, and significance was determined by two-sided *t* test. *, P < 0.05; **, P < 0.01; ****, P < 0.0001. Source data are available for this figure: SourceData F1.

**Figure S1.**
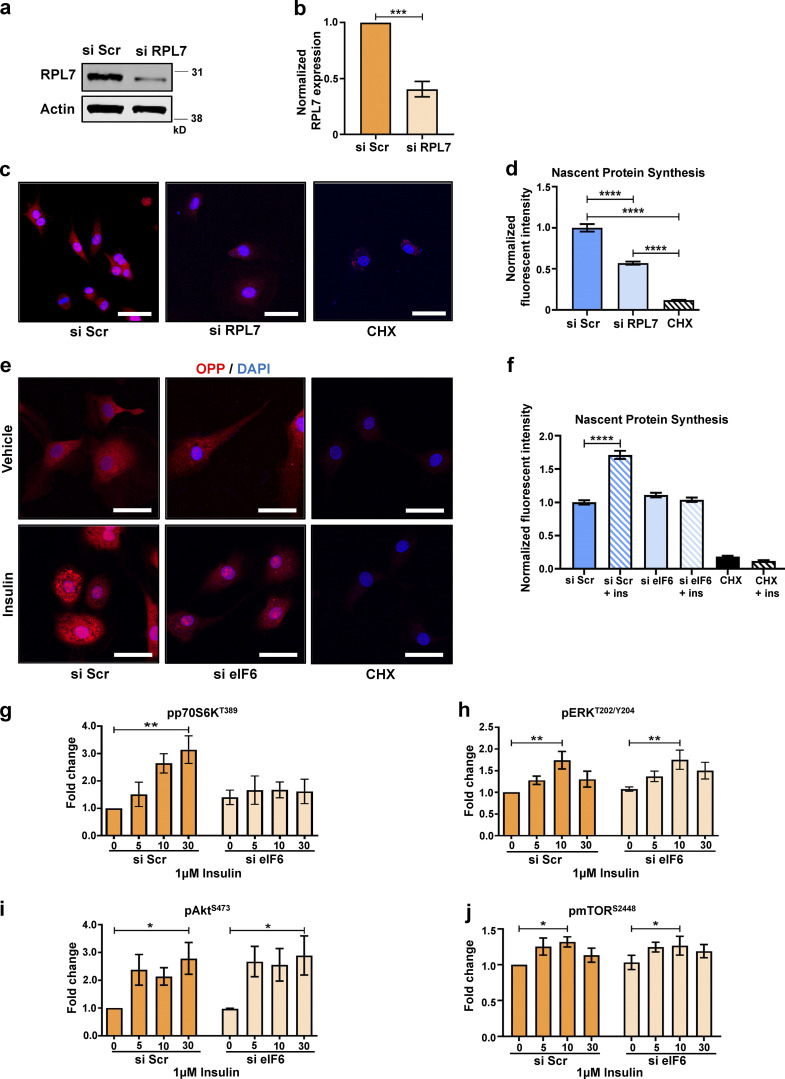
**Contribution of eIF6 and ribosomal proteins to nascent protein synthesis.** Cells were transfected with si Scr or si RPL7. **(a and b) **Representative Western blot (a) and mean quantification (b) of siRNA knockdown of RPL7 (*n* = 3). **(c–f)** OPP was incorporated into cells to label nascent proteins (red) using a Click-iT assay, and cell nuclei were costained with DAPI (blue). Cycloheximide (CHX) was used as a negative control for OPP incorporation. **(c)** Representative fluorescent micrographs of si Scr– or si RPL7–transfected ECs or CHX-treated ECs. Scale bars = 20 μm. **(d)** Quantification of cell fluorescence following OPP incorporation Click-iT assay in c (*n* > 30 cells across three separate experiments). **(e)** Representative fluorescent micrographs of si Scr– or si eIF6–transfected ECs or CHX-treated ECs, ±30-min insulin stimulation. Scale bars = 20 μm. **(f)** Quantification of cell fluorescence following OPP incorporation Click-iT assay in e (*n* > 30 cells across three separate experiments). **(g–j)** Quantification of phosphorylated p70S6K (pp70S6K^T389^) relative to p70S6K (g), pERK^T202/Y204^ relative to ERK (h), pAkt^S473^ relative to Akt (i), and pmTOR^S2448^ relative to mTOR (j) in response to 1 μM insulin (for 0, 5, 10, or 30 min) in si Scr or si eIF6 A431 cells (*n* = 3). Values in b, d, and f–j are mean ± SEM, and significance in b was determined by two-sided *t* test and in d and f–j by two-way ANOVA. *, P < 0.05; **, P < 0.01; ***, P < 0.001; ****, P < 0.0001. ins, insulin. Source data are available for this figure: SourceData FS1.

The role of eIF6 in ECs has not been investigated. We transfected primary ECs with scrambled (Scr) or eIF6 siRNAs (si Scr or si eIF6, respectively) and examined protein synthesis and ribosomal biogenesis. After confirming effective knockdown of >90% (Fig. 1, a and b), we used O-propargyl-puromycin (OPP) to label nascent proteins in control and eIF6-depleted cells. OPP contains an alkyne group, which through Click chemistry, can be covalently coupled to fluorescent tags for visualization of nascent proteins (Signer et al., 2014). Despite almost complete knockdown of eIF6, unstimulated eIF6-depleted cells did not display defects in nascent protein synthesis (Fig. 1, c and d). This observation in ECs is consistent with previous reports showing that loss of eIF6 does not affect basal protein synthesis in eIF6 haploinsufficient hepatocytes and skeletal muscle cells as well as in eIF6 siRNA-transfected fibroblasts and HeLa cells (Brina et al., 2015a; Chendrimada et al., 2007; Clarke et al., 2017; Gandin et al., 2008). To test that our cells were behaving correctly, we monitored nascent protein synthesis in ECs in which the ribosomal protein RPL7, a key constituent of the large 60S subunit and active 80S monosomes, had been knocked down; these cells showed a dramatic reduction in puromycin incorporation and, consequently, a significant decrease in nascent protein synthesis (Fig. S1, a–d). To complement the puromycin incorporation assays and further test the role of eIF6 in protein synthesis, we used ultracentrifugation of cytoplasmic extracts from si Scr and si eIF6 cells through sucrose gradients to fractionate the large RNP complexes involved in protein translation. With this technique, the physically separated ribosome-containing RNP complexes produce a polysome profile when observed by UV light at 254 nm and fractionated, allowing the efficiency of active protein translation in a cell sample to be evaluated. In agreement with previous findings (Brina et al., 2015a; Chendrimada et al., 2007; Clarke et al., 2017; Gandin et al., 2008), we found no change in the general profile of the polysome peaks, indicative of no visible defects in protein translation efficiency (Fig. 1 e). This finding is consistent with our results showing no defects in nascent protein synthesis in unstimulated eIF6-depleted cells. Although loss of eIF6 does not cause basal defects in protein translation, eIF6 has been reported to be required for efficient protein translation in response to insulin stimulation (Brina et al., 2015a; Miluzio et al., 2016). To test if this is also true in our system, we assessed nascent protein synthesis and associated signaling (Roux and Topisirovic, 2018) in response to insulin in control and eIF6-depleted cells. In agreement with previous studies (Brina et al., 2015a; Gandin et al., 2008; Miluzio et al., 2016), we found that insulin-induced nascent protein synthesis was abrogated in eIF6-depleted cells (Fig. S1, e and f), with corresponding reductions in activation of p70S6K (Fig. S1 g). However, activation of ERK1/2, Akt, and mammalian target of rapamycin (mTOR) were unaffected with loss of eIF6 (Fig. S1, h–j), again consistent with previous reports.

Another previously described role of eIF6 is in ribosomal biogenesis (Basu et al., 2001; Brina et al., 2015a). To assess for possible ribosomal biogenesis defects in our system, we used a multipronged approach. First, we assayed nucleolar stress by quantifying nucleolar size and number in control and si eIF6–transfected cells. Confocal microscopy of nucleolin staining revealed a small decrease in nucleolar number and a decrease in nucleolar size in eIF6-depleted cells (Fig. 1, f–h). We then sought to see if this apparent nucleolar stress manifested as a defect in precursor ribosomal RNA (pre-rRNA) levels by quantitative PCR (qPCR). We found no differences in any of the transcripts we measured (*45S*, *28S*, *18S*, and *5.8S*) despite ∼90% knockdown efficiency (Fig. 1, i–l). Finally, we examined protein expression levels of four key 60S ribosomal proteins, RPL7a, RPL10a, RPL26, and RPL23, which revealed small or no detectable differences following loss of eIF6 (Fig. 1, m–q). Given that the half-life of ribosomes is ∼5 d (Hirsch and Hiatt, 1966; Nikolov et al., 1987) and all our experiments are performed within a 48–72-h window of siRNA-mediated knockdown, it is perhaps unsurprising that levels of ribosomal proteins are relatively unaffected by loss of eIF6. Taken together, these results show that although eIF6 depletion causes mild nucleolar stress, there are minimal eIF6-dependent changes in pre-rRNA, ribosomal protein expression, and nascent protein synthesis (as assayed by Click-iT and polysome profiles) in our system. These results are consistent with previous studies that showed that eIF6 siRNA-mediated knockdown in mammalian cells maintains protein synthesis and ribosomal biogenesis (Gandin et al., 2008).

### Endogenous eIF6 regulates cytoskeletal organization and cell-mediated forces

**Figure 2.**
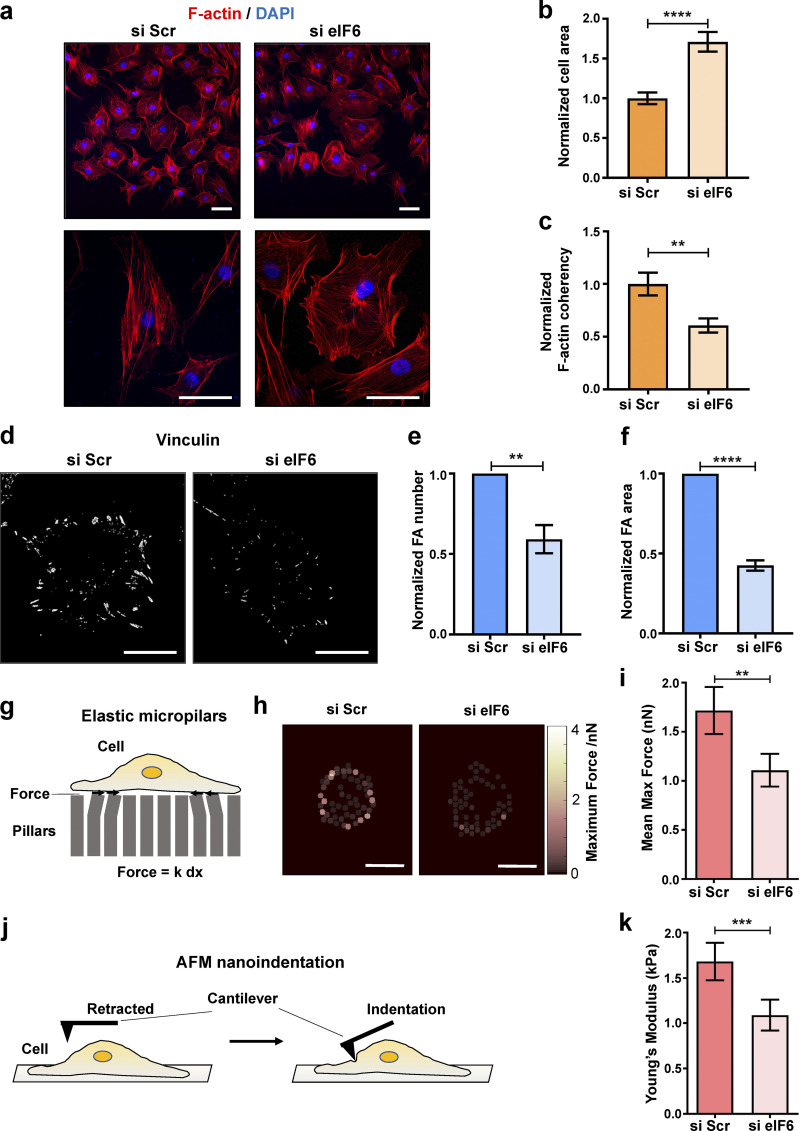
**eIF6 regulates cell-generated forces. (a)** Representative immunofluorescent micrographs showing F-actin (phalloidin; red) and nuclei (DAPI, blue) in ECs transfected with si Scr or si eIF6 imaged at ×20 (top) and ×63 (bottom). Scale bars = 50 μm and 20 μm, respectively. **(b and c)** Quantification of mean cell area (b) and coherency in alignment of F-actin fibers (c) in si Scr and si eIF6 cells (*n* > 30 cells across three separate experiments). **(d)** Representative immunofluorescent micrographs showing vinculin-positive focal adhesions (white) in si Scr and si eIF6 ECs. Scale bars = 20 μm. **(e and f)** Quantification of mean number (e) and mean area (f) of vinculin-positive focal adhesions (*n* > 30 cells across three separate experiments). **(g–k)** Traction force microscopy and AFM measurements in si Scr and si eIF6 ECs (schematic representations shown in g and j, respectively). Force vector maps (h) indicate the magnitude of traction forces calculated from maximum pillar displacement. Quantification of mean micropillar displacement (i) and Young’s modulus (k; *n* > 30 cells across three separate experiments). Values in b, c, e, f, i, and k are mean ± SEM, and significance was determined by two-sided *t* test. **, P < 0.01; ***, P < 0.001; ****, P < 0.0001. FA, focal adhesion; max, maximum.

**Figure S2.**
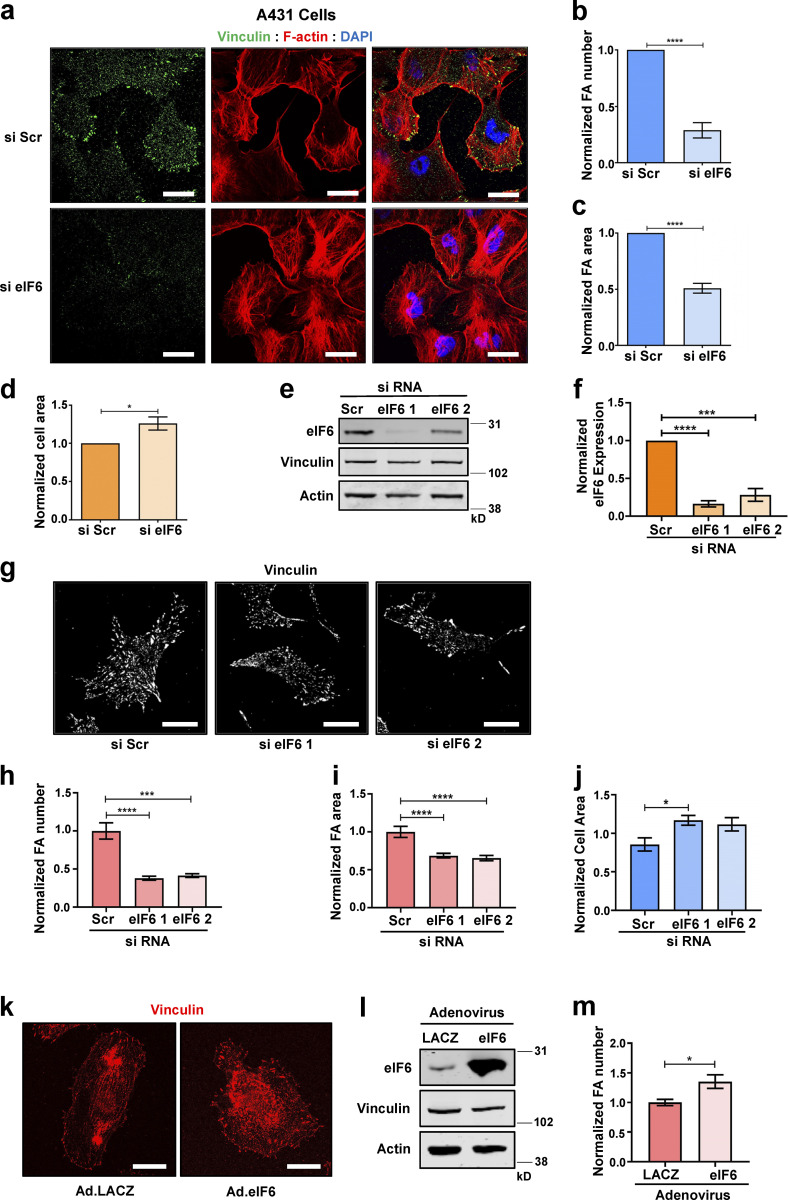
**eIF6 regulates focal adhesions. (a–d)** A431 cells were transfected with si Scr or si eIF6. **(a)** Representative immunofluorescent micrographs of cells showing focal adhesions (vinculin; green), filamentous actin (phalloidin; red), and nuclei (DAPI; blue). Scale bars = 20 μm. **(b and c)** Quantification of mean frequency of focal adhesions per cell (b) and focal adhesion area (c; *n* > 30 cells across three separate experiments). **(d)** Quantification of cell area (*n* > 30 cells across three separate experiments). **(e–j)** ECs were transfected with si Scr or one of two individual eIF6 siRNAs (si eIF6 1 and si eIF6 2). **(e and f)** Representative Western blot for eIF6 protein levels (e) and quantification of Western blot band intensities (f; *n* = 3). **(g)** Representative immunofluorescent micrographs showing vinculin-positive focal adhesions (white) in si Scr, si eIF6 1, and si eIF6 2 cells. Scale bars = 20 μm. **(h and i)** Quantification of mean frequency per cell (h) and mean area (i) of vinculin-positive focal adhesions (*n* >30 cells across three separate experiments). **(j)** Quantification of mean cell area (*n* > 30 cells across three separate experiments). **(k–m)** ECs were transduced with control adenovirus (Ad.LACZ) or an eIF6-expressing adenovirus (Ad.eIF6). **(k)** Representative immunofluorescent micrographs showing vinculin-positive focal adhesions (red). Scale bars = 20 μm. **(l)** Representative Western blot for eIF6 protein level (*n* = 5). **(m)** Quantification of mean frequency of vinculin-positive focal adhesions per cell (*n* > 30 cells across three separate experiments). Values in b–d, f, h–j, and m are mean ± SEM, and significance was determined by two-way *t* test; *, P < 0.05; ***, P < 0.001; ****, P < 0.0001. FA, focal adhesion. Source data are available for this figure: SourceData FS2.

Despite the lack of obvious protein synthesis defects under steady-state conditions, examination of cells transfected with si eIF6 revealed that cells depleted of eIF6 displayed altered overall cell morphology and increased cell-surface area (Fig. 2, a and b). Cells with reduced eIF6 displayed disorganized F-actin, as assayed by a reduction in orientation coherency (ranging from 0 to 1; Fig. 2, a and c). To determine if eIF6 is also involved in mechanosensitive focal adhesions, we quantified the number and size of vinculin-positive focal adhesions and showed that eIF6-depleted cells displayed significantly smaller and fewer vinculin-positive focal adhesion complexes relative to eIF6-expressing cells (Fig. 2, d–f). Similar results were obtained when we examined focal adhesions in human epidermoid carcinoma (A431) cells (Fig. S2, a–d). As additional controls for off-target effects of siRNA, we also tested two single siRNAs for eIF6, which displayed the same phenotypes (Fig. S2, e–j).

**Figure S3.**
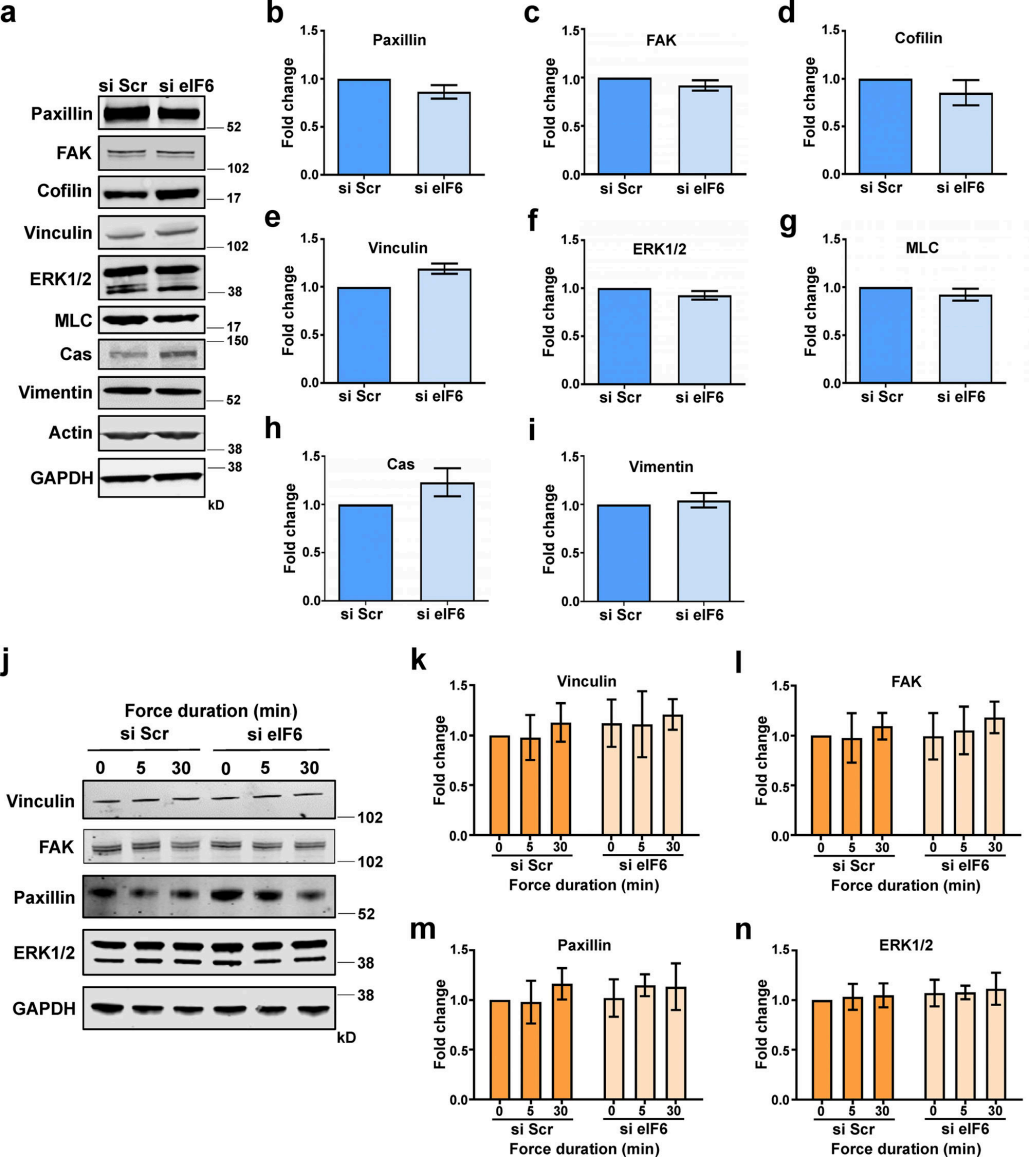
**Depletion of eIF6 does not affect cytoskeletal or focal adhesion protein levels. (a)** Representative Western blots for cytoskeletal proteins from si Scr– or si eIF6–transfected EC lysates. **(b–i)** Quantification of band intensity for Western blot analysis for paxillin (b), FAK (c), cofilin (d), vinculin (e), ERK1/2 (f), myosin light chain (MLC; g), Crk-associated substrate p130^cas^ (Cas; h), and vimentin (i; *n* > 3). **(j–n)** ECs were transfected with si Scr or si eIF6 and subjected to force application on PECAM-1. **(j)** Representative Western blots for cytoskeletal proteins vinculin, FAK, paxillin, and ERK1/2. **(k–n)** Quantification of mean band intensity for Western blot analysis for vinculin (k), FAK (l), paxillin (m), and ERK1/2 (n) compared with loading control GAPDH (*n* > 3). Values in b–i and k–n are mean ± SEM, and significance was determined in b–h by two-sided *t* test and in i and k–n by two-way ANOVA. Source data are available for this figure: SourceData FS3.

To explore whether the cytoskeleton phenotype of eIF6-depleted cells was related to changes in protein expression, we measured expression of a subset of key cytoskeleton proteins in our cell lysates. In agreement with the Click-iT assay and polysome profiles, total expression levels of cytoskeletal proteins were unaffected by knockdown of eIF6 (Fig. S3, a–i). Taken together, these results show that regulation of cell mechanics by eIF6 is likely not a by-product of reduced nascent protein synthesis or altered cytoskeletal protein levels.

In addition to this loss-of-function approach, we examined the effects of gain of function by overexpression of eIF6; we found that overexpression of eIF6 gave a small, but statistically significant, increase in the number of vinculin-positive focal adhesions (Fig. S2, k–m). Together, these results suggest that eIF6 plays a regulatory role in the organization of the structural components of the cell.

The cytoskeleton is a fundamental and highly dynamic structure that regulates the mechanical properties of the cell via transmission of force (Wang et al., 1993). Given the effects of eIF6 silencing on both actin stress fibers and focal adhesions, we assessed endogenous force generation and stiffness of eIF6-depleted cells. To investigate if loss of eIF6 leads to changes in the forces cells exert on their substrate, ECs were grown on a substrate consisting of an array of elastic polydimethylsiloxane micropillars coated with fibronectin (Chronopoulos et al., 2016). The deflection of each pillar is proportional to cell traction force and was optically monitored (Fig. 2 g). si Scr–transfected cells generated traction stresses that were mostly concentrated around the periphery (Fig. 2 h). In contrast, eIF6-depleted cells showed a marked decrease in the overall traction force generation (Fig. 2, h and i).

The capacity for force application on substrates is intimately connected to cytoskeletal stiffness (Kraning-Rush et al., 2011; Stamenović, 2005). Using atomic force microscopy (AFM), we measured the surface stiffness of individual ECs by nanoindentation (Chronopoulos et al., 2016) following si Scr or si eIF6 transfection (Fig. 2 j). We ensured that our analysis would assess the contribution of the cytoskeleton to cell compliance by indenting the cells at points between the nucleus and the cell edges. We observed that loss of eIF6 resulted in reduced Young’s modulus and, thus, reduced cellular stiffness (Fig. 2 k). Collectively, these results suggest that eIF6, via regulation of the cytoskeleton, determine endogenous force generation and stiffness of cells.

### eIF6 is required for the cellular response to external mechanical force

**Figure 3.**
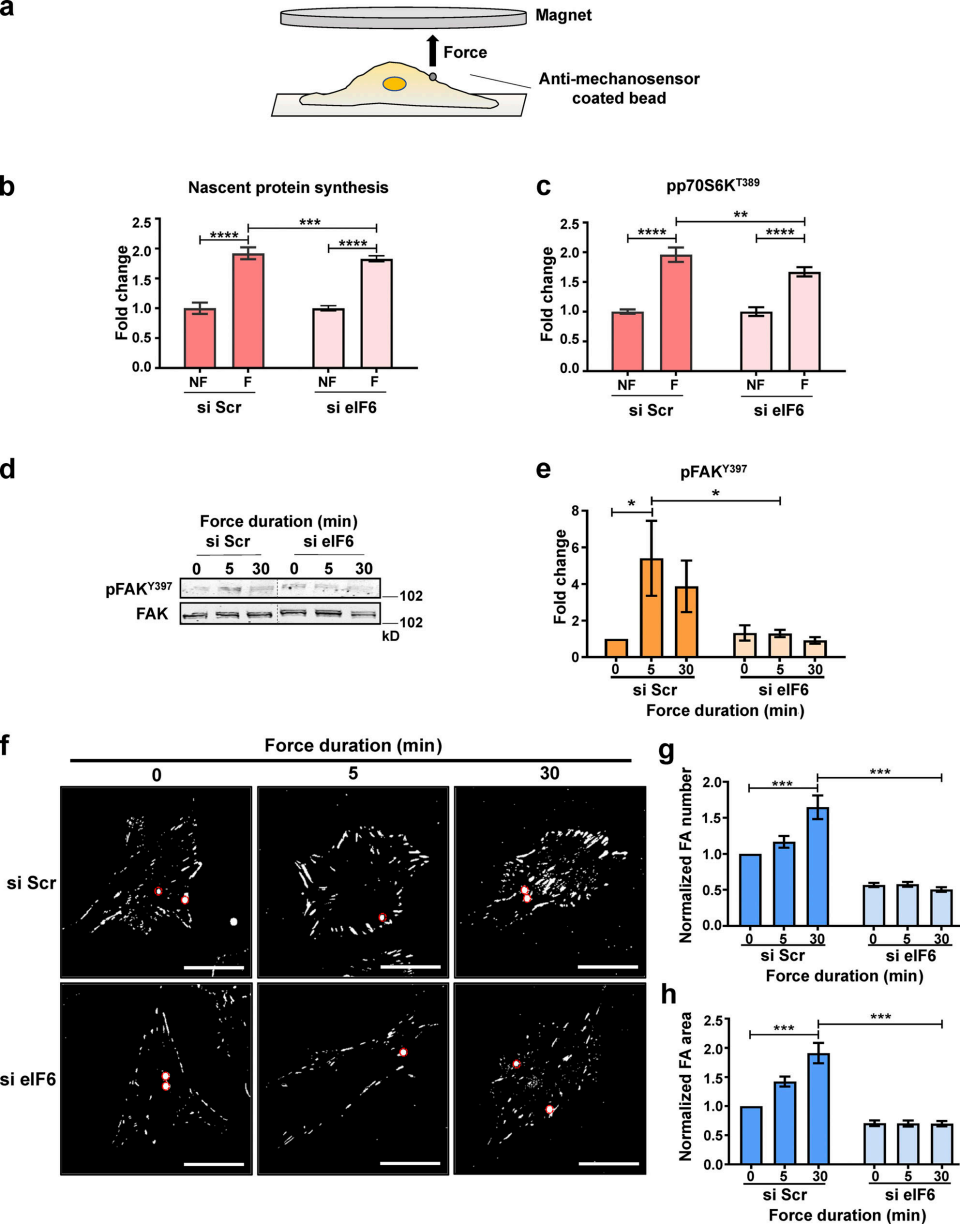
**Decoupling of eIF6 cellular functions. (a)** Schematic representation of permanent magnet system used to apply direct force on cellular mechanosensors. **(b and c)** Mechanical force was applied for 0 min (no force [NF]) or 30 min (force [F]) to cells transfected with si Scr or si eIF6 and incubated with puromycin. Quantification of mean fluorescence of puromycin (b) and phosphorylated p70S6K (pp70S6K^T389^; c; *n* > 30 cells across three separate experiments). **(d–h)** si Scr and si eIF6 ECs were exposed to mechanical force for 0, 5, or 30 min. **(d and e)** Representative Western blots of phosphorylated FAK (pFAK^Y397^) and total FAK protein levels from EC lysates (d) and quantification of band intensity (e; *n* = 4). **(f)** Representative immunofluorescent micrographs showing focal adhesions (vinculin; white) in ECs following force. Magnetic beads are highlighted by red circles. Scale bars = 20 μm. **(g and h)** Quantification of mean frequency per cell (g) and mean area (h) of vinculin-positive focal adhesions (*n* > 30 cells across three separate experiments). Values in b, c, e, g, and h are mean ± SEM, and significance was determined by two-way ANOVA. *, P < 0.05; **, P < 0.01; ***, P < 0.001; ****P < 0.0001. Source data are available for this figure: SourceData F3.

**Figure S4.**
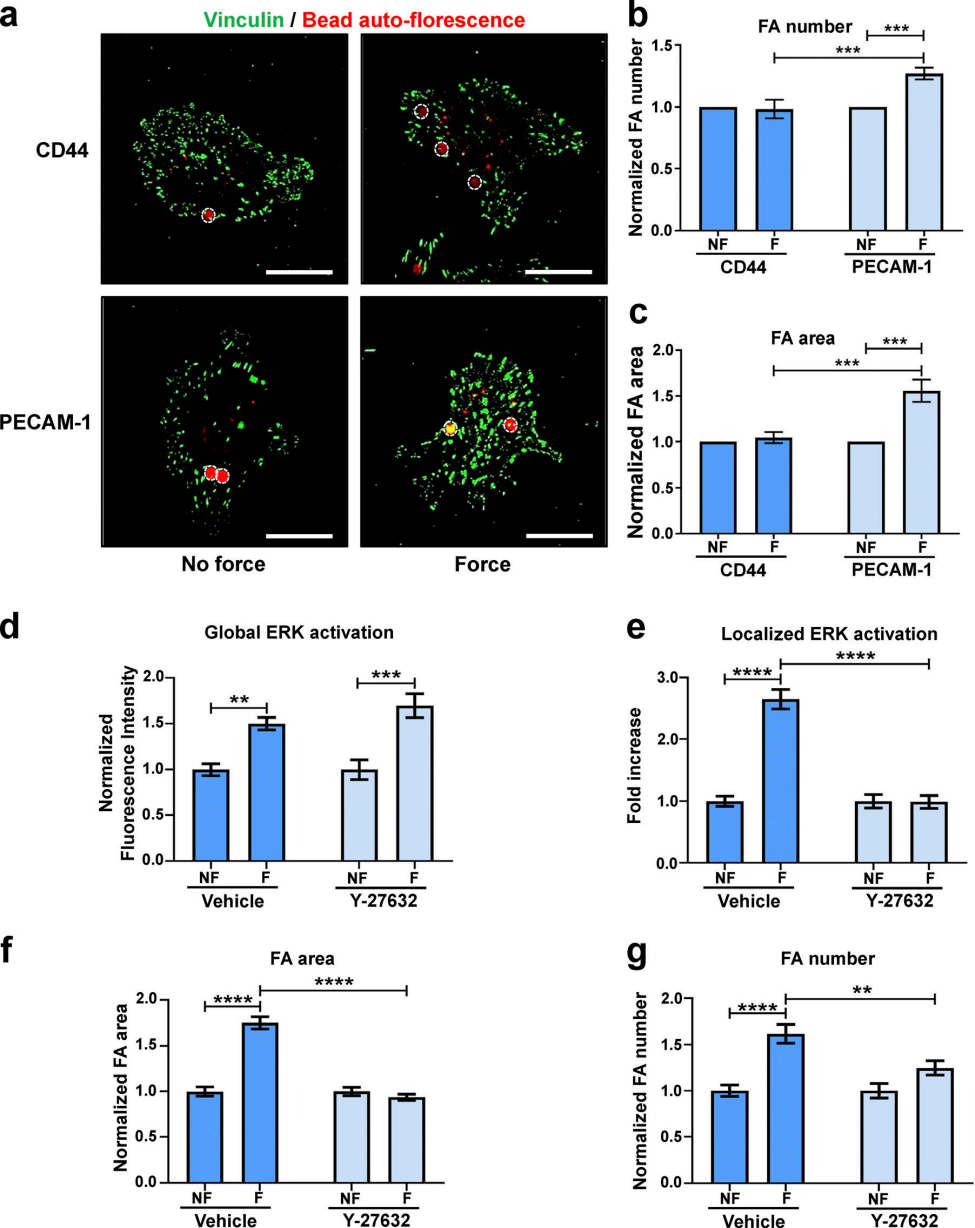
**Tensional force on PECAM-1 elicits global focal adhesion growth that is dependent on cellular contractility. (a–c)** ECs were incubated with magnetic beads coated with an antibody specific to CD44 or PECAM-1 and subjected to force for 0 min (no force [NF]) or 30 min (force [F]) with a permanent magnet. **(a)** Representative fluorescent micrographs of cells showing focal adhesions (vinculin; green) with bound beads (red, marked by white circles). Scale bars = 20 μm. **(b and c)** Quantification of mean frequency of focal adhesions per cell (b) and mean focal adhesion area (c) following NF or F (*n* > 30 cells across three separate experiments). **(d–g)** ECs were treated with vehicle or ROCK inhibitor Y-27632, and then mechanical force was applied for 0 min (NF) or 30 min (F). Quantification of global activation of ERK (d), colocalization of pERK1/2^T202/Y204^ to vinculin (e) quantified using Pearson’s coefficient, mean area of vinculin-positive focal adhesions (f), and mean frequency of focal adhesions per cell (g; *n* > 30 cells across three separate experiments). Values in b–g are mean ± SEM, and significance was determined in b and c by two-sided *t* test and in d–g by two-way ANOVA. **, P < 0.01; ***, P < 0.001; ****, P < 0.0001. FA, focal adhesion.

In addition to endogenous forces generated when a cell pulls on the ECM via the cytoskeleton, cells remodel their cytoskeleton and focal adhesions in response to externally applied forces. There is a dynamic feedback system that allows coupling of externally applied forces to internal forces via mechanosignaling (Chen, 2008). In ECs specifically, application of localized tensional forces on PECAM-1, a transmembrane mechanosensor, initiates mechanosignaling cascades that ultimately result in cell-wide growth of focal adhesions and changes in cytoskeletal architecture (Collins et al., 2012, 2014); this cell-wide focal adhesion growth is a property unique to PECAM-1 and does not occur when force is applied to integrins. Having identified a role for eIF6 in endogenous force generation that results in focal adhesion homeostasis (Fig. 2), we next determined if eIF6 is also required for exogenous force transduction. For this, we used a well-established approach (Collins et al., 2012, 2014) to directly apply force to PECAM-1 (Fig. 3 a). Magnetic beads were coated with an antibody against the extracellular domain of PECAM-1 (or CD44 as a control; Fig. S4, a–c) and allowed to bind to ECs before a constant force (∼10 pN) was applied for the indicated times using a magnet.

We first tested the role of eIF6 in nascent protein synthesis in response to mechanical force by examining puromycin incorporation (Fig. 3 b). Similar to our findings in Fig. 1 (c and d), quantification of puromycin revealed that both control and eIF6-depleted ECs had the same basal levels of nascent protein synthesis. Importantly, both control and eIF6-depleted ECs responded to mechanical force application by increasing nascent protein synthesis, although there was a very slight decrease in knockdown cells (Fig. 3 b). To corroborate these results, we also assayed activation of the p70S6K pathway. Similar to the puromycin result, we noticed increased phosphorylation of p70S6K in both si Scr– and si eIF6–transfected cells in response to force (Fig. 3 c), thus providing solid evidence that loss of eIF6 does not disrupt global protein synthesis pathways in response to force.

We then asked if eIF6 is involved in force-induced mechanical signaling and focal adhesion growth. Using the same bead-pulling assay, we assayed phosphorylation of FAK at tyrosine (Y)397, as this site is phosphorylated under conditions where adhesions are growing in response to an increase in force (Seufferlein and Rozengurt, 1994; Sinnett-Smith et al., 1993). Our results showed that loss of eIF6 abrogated force-induced phosphorylation of FAK at Y397, suggesting defects in the activation of this important mediator of mechanotransduction pathways (Fig. 3, d and e). Consistent with this result, we observed that while cells expressing eIF6 exhibited robust focal adhesion growth in both size and number in response to force, eIF6-depleted cells not only showed basal defects in focal adhesion number and size, consistent with Fig. 2, but also failed to respond to force, as they did not increase focal adhesion size or number (Fig. 3, f–h). Taken together, these results show that eIF6 triggers dynamic activation of force-driven mechanical pathways and downstream cell-wide focal adhesion growth.

**Figure 4.**
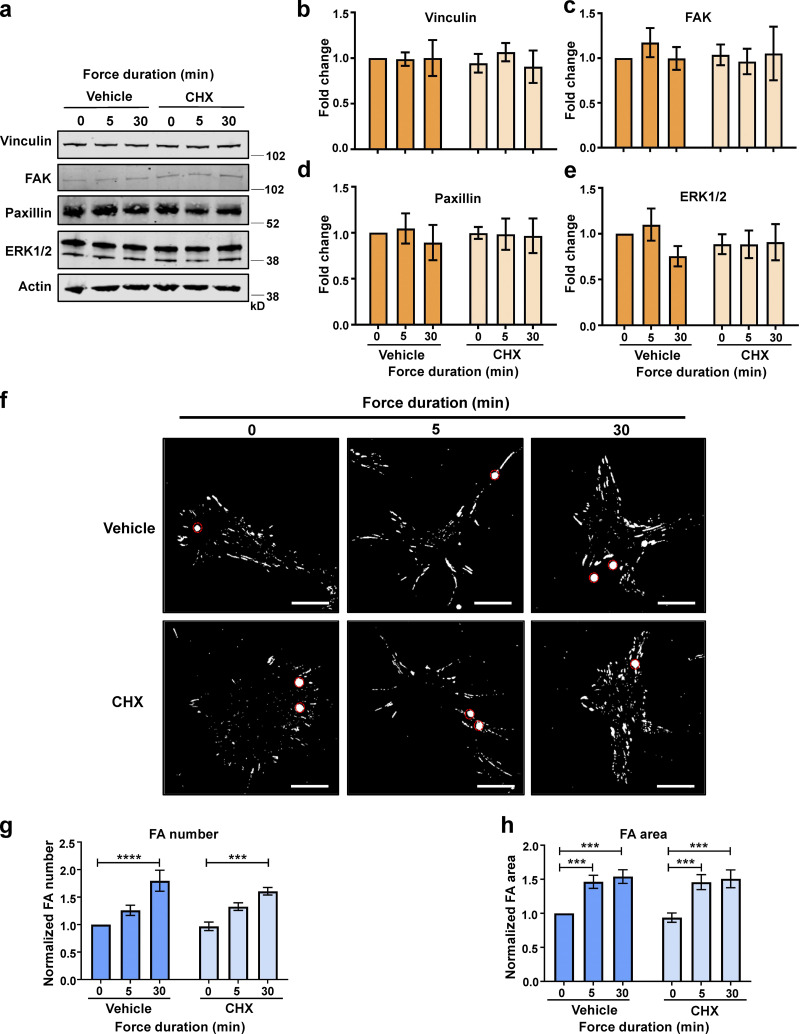
**Force-induced focal adhesion growth is independent of protein synthesis.** ECs were pretreated with DMSO (vehicle) or DMSO + cycloheximide (CHX) and exposed to force on PECAM-1 for 0, 5, or 30 min using a permanent magnet in the presence of vehicle or CHX. **(a)** Representative Western blots of cytoskeletal proteins from EC lysates following force. **(b–e)** Quantification of vinculin (b), FAK (c), paxillin (d), and ERK1/2 (e) relative to actin loading control (*n* > 3). **(f)** Representative immunofluorescent micrographs showing focal adhesions (vinculin; white) in ECs following force. PECAM-1–coated beads are highlighted by red circles. Scale bars = 20 μm. **(g and h)** Quantification of mean frequency per cell (g) and mean area (h) of vinculin-positive focal adhesions (*n* > 40 cells across three separate experiments). Values in b–e, g, and h are mean ± SEM, and significance was determined by two-way ANOVA. ***, P < 0.001; ****, P < 0.0001. FA, focal adhesion. Source data are available for this figure: SourceData F4.

Our data so far have shown that while force-induced increase in nascent protein synthesis and associated signaling does not require eIF6, the global increase in focal adhesions is eIF6 dependent. To examine this differential requirement for eIF6 in translation versus cytoskeleton remodeling, we first determined if force application affected the expression levels of key cytoskeletal proteins in response to force. Quantification showed no changes in total levels of vinculin, FAK, or paxillin following force application in either group and no differences in the levels of cytoskeletal proteins following depletion of eIF6 (Fig. S3, j–m), suggesting that neither force application nor depletion of eIF6 affects expression levels of focal adhesion proteins. To investigate this further, we examined the force response in the presence of the protein synthesis inhibitor cycloheximide; protein levels of vinculin, FAK, paxillin, or the signaling mediator ERK1/2 were unaffected by force or in response to cycloheximide (Fig. 4, a–e). We then examined the effect of cycloheximide in force-induced focal adhesion growth. Our results showed that cells increased vinculin-positive focal adhesion size and number despite the presence of cycloheximide (Fig. 4, f–h), further demonstrating that new protein synthesis is not required for the rapid, force-responsive assembly of focal adhesions. Importantly, we also found no differences between nonforce conditions, showing that cycloheximide did not affect focal adhesions in the absence of force.

Taken together, these results allow, for the first time, uncoupling of the mechanical and translational functions of eIF6 and show that eIF6 is required for the cell-wide focal adhesion growth in response to force without significantly affecting protein synthesis responses.

### eIF6-mediated tension regulates spatial activation of the ERK1/2 pathway

**Figure 5.**
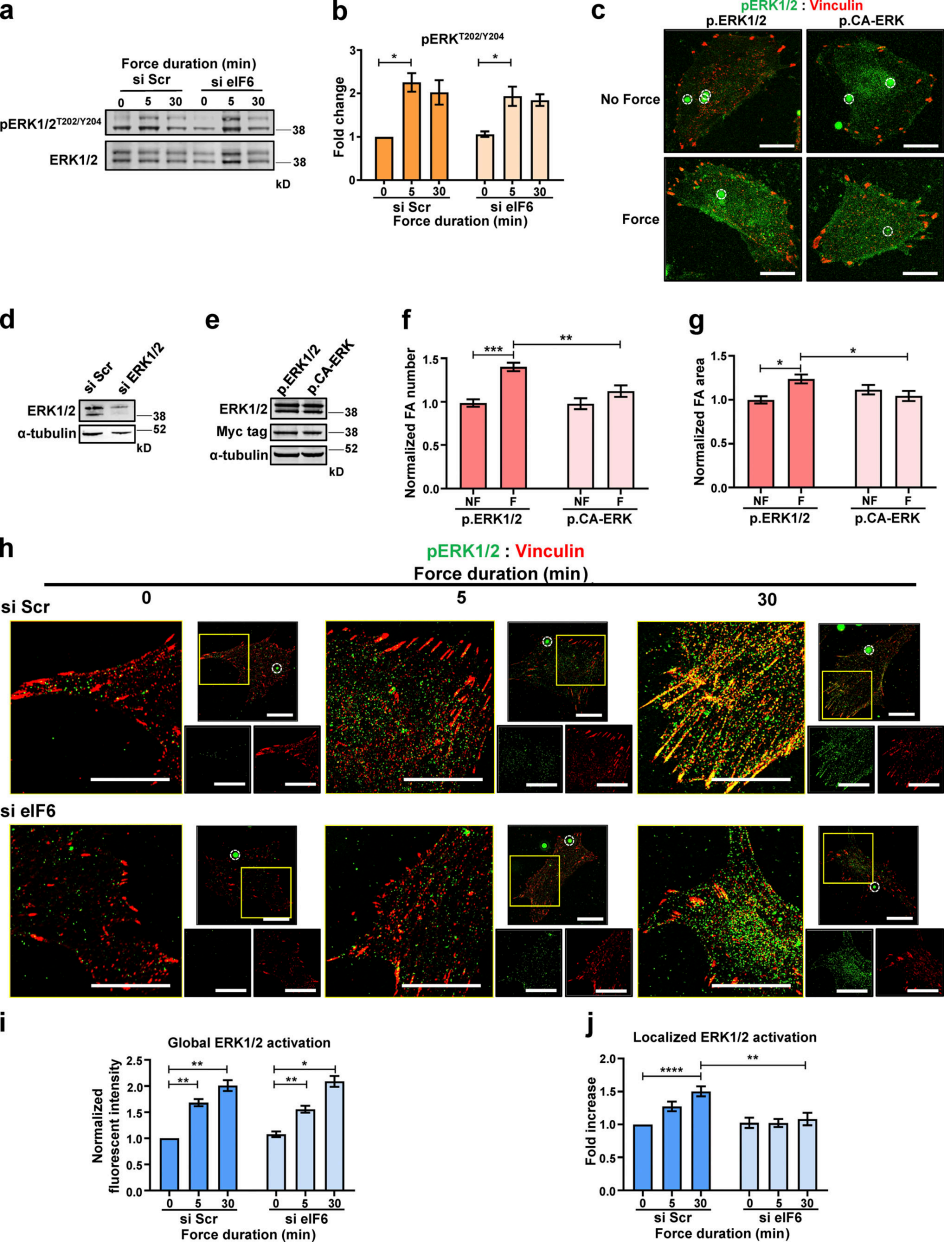
**eIF6 regulates the spatial mechanoactivation of ERK1/2.** ECs transfected with si Scr or si eIF6 were exposed to force for 0, 5, or 30 min. **(a and b)** Representative Western blots of pERK^T202/Y204^ and t-ERK1/2 and quantification (*n* = 4). **(c–e) **ECs were transfected with si Scr or si ERK1/2 (d), and following siRNA-mediated knockdown, ECs were transiently transfected with plasmids encoding WT ERK1/2 (p.ERK1/2) or CA-ERK (p.CA-ERK; e). **(c, f, and g)** Mechanical force was applied for 0 min (no force [NF]) or 30 min (force [F]) to p.ERK1/2- and p.CA-ERK–expressing ECs. Representative superresolution immunofluorescent micrographs showing pERK^T202/Y204^ (green) and focal adhesions (vinculin; red) in ECs following force (c); PECAM-1–coated beads are highlighted by white circles. Scale bars = 20 μm. Quantification of mean frequency per cell (f) and mean area (g) of vinculin-positive focal adhesions following force for 0 min (NF) or 30 min (F; *n* > 30 cells across three separate experiments). **(h)** Representative superresolution immunofluorescent micrographs showing colocalization of pERK^T202/Y204^ (green) with focal adhesions (vinculin, red) following application of force. Larger images are higher magnification images of indicated region of whole cells shown in smaller images. Magnetic beads are highlighted by white circles. Scale bars = 20 μm. **(i)** Quantification of mean fluorescence intensities of pERK^T202/Y204^ following application of force (*n* > 30 cells across three separate experiments). **(j)** Image analysis quantification of colocalization of pERK^T202/Y204^ with focal adhesions, using Pearson’s coefficient, following force (*n* > 30 cells across three separate experiments). Values in b, f, g, i, and j are mean ± SEM, and significance was determined by two-way ANOVA. *, P < 0.05; **, P < 0.01; ****, P < 0.0001. FA, focal adhesion. Source data are available for this figure: SourceData F5.

Having shown that eIF6 regulates mechanical responses without significant effects on protein synthesis, we sought to determine the molecular mechanisms of this mechanoregulation. A prominent mechanosensitive pathway known to be regulated by both intracellular and extracellular mechanical cues is the ERK signaling cascade. Importantly, published work has demonstrated force-dependent activation of the ERK1/2 pathway downstream of mechanical tension on PECAM-1 (Chiu et al., 2008; Chrétien et al., 2010; Collins et al., 2012; Osawa et al., 2002). We therefore asked if eIF6 regulates the mechanoactivation of ERK1/2. Similar to previous reports, application of force on PECAM-1 induced activation of ERK1/2 in si Scr cells, as assayed by increased phosphorylation at threonine (T)202 and Y204 (pERK^T202/Y204^; Fig. 5, a and b). Surprisingly, despite defective force-induced focal adhesion growth, eIF6-depleted cells showed normal levels of pERK^T202/Y204^ at baseline and in response to force, similar to those seen in control cells (Fig. 5, a and b). Total ERK1/2 expression levels were also unchanged with loss of eIF6 (Fig. S3, j and n). On first look, these results suggested that the defect in force-induced focal adhesion growth does not lie with ERK1/2. However, we also considered possible differences in the spatial activation of the ERK1/2 pathway, as active ERK1/2 has been reported to localize to the actin cytoskeleton and focal adhesions, and the correct spatial localization of active ERK1/2 is important for downstream cytoskeletal remodeling (Appel et al., 2010; Fincham et al., 2000; Pritchard et al., 2004; Vetterkind et al., 2012, 2013; Zuckerbraun et al., 2003). To examine if localized ERK1/2 activity at focal adhesions is required for force-induced focal adhesion remodeling, we transfected cells with plasmids encoding either WT ERK1/2 or a constitutively active ERK–MEK fusion protein (CA-ERK; Robinson et al., 1998). This form of ERK protein is constitutively active globally and does not polarize to focal adhesions (Fig. 5 c; Robinson et al., 1998). After knockdown of endogenous ERK1 and ERK2 using siRNAs (Fig. 5 d), we reexpressed either WT ERK1 and ERK2 or CA-ERK (Fig. 5 e) and examined force-induced focal adhesion remodeling. We found that in agreement with Fig. 3, f–h, WT ERK1/2-expressing cells responded to force by increasing both focal adhesion size and number (Fig. 5, c, f, and g). However, CA-ERK–expressing cells failed to increase focal adhesion size and/or number in response to force (Fig. 5, c, f, and g), thus demonstrating that localized ERK activation at focal adhesions is critical for force-induced focal adhesion remodeling. Importantly, we also found no differences between nonforce conditions, showing that CA-ERK did not affect focal adhesions in the absence of force.

**Figure S5.**
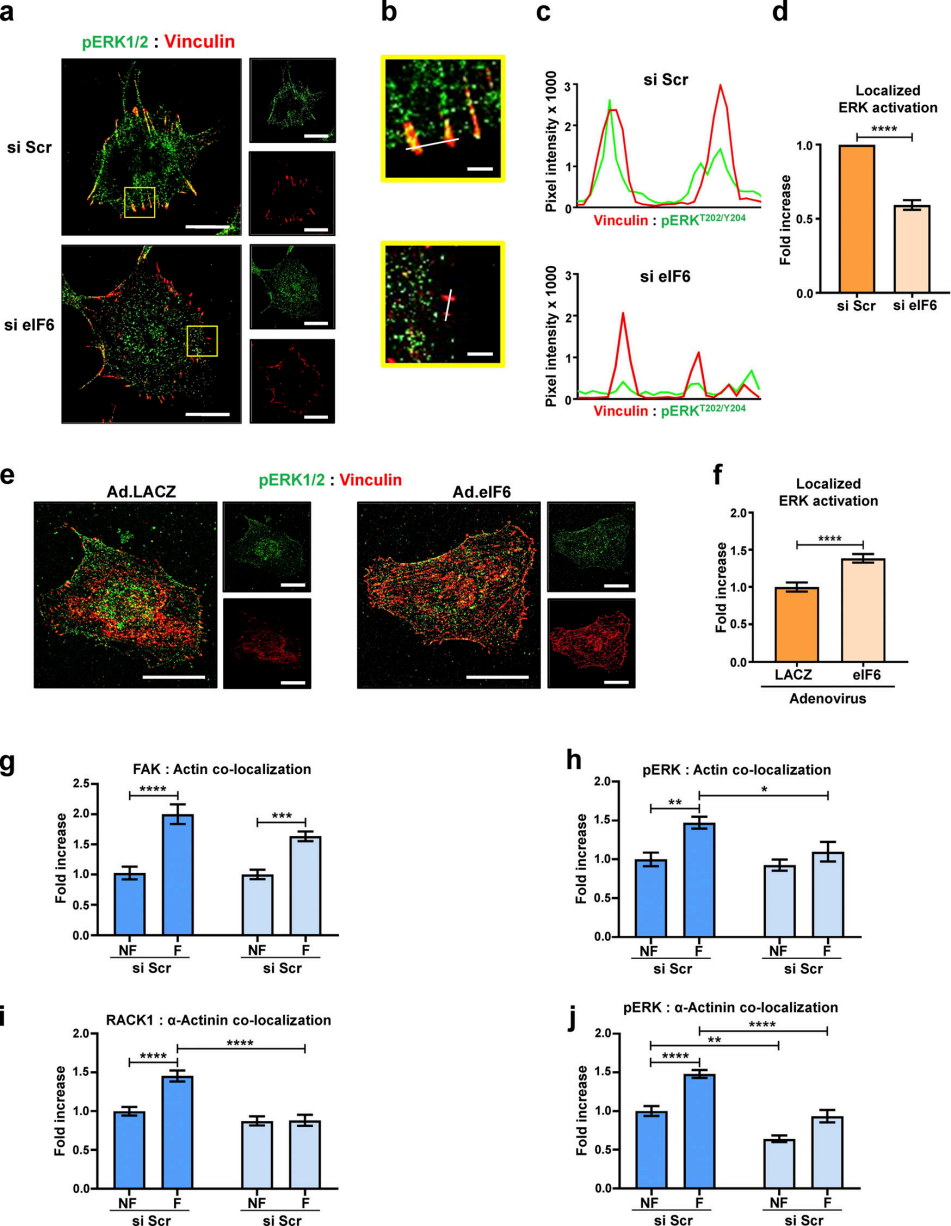
**eIF6 regulates localization of activated ERK1/2 and mechanocomplex formation at focal adhesions. (a–d)** Depletion of eIF6 disrupts localization of activated ERK1/2 in migrating cells. **(a)** Representative immunofluorescent confocal micrographs for si Scr– and si eIF6–transfected ECs showing pERK1/2^T202/Y204^ (green) and vinculin (red). Scale bars = 20 μm. **(b)** Higher magnification images of selected region in a. Scale bars = 2 μm. **(c)** Representative fluorescent intensities along the white line indicated in the merged image shown in b were quantified using line scan mode. Line scans are plotted in the graph shown. **(d)** Colocalization of pERK1/2^T202/Y204^ to vinculin was quantified using Pearson’s coefficient (*n* > 30 cells across three separate experiments). **(e and f)** Overexpression of eIF6 increases localization of activated ERK1/2 at focal adhesions. **(e)** Representative immunofluorescent confocal micrographs for ECs transduced with control adenovirus (Ad.LACZ) or an eIF6-expressing adenovirus (Ad.eIF6) showing pERK1/2^T202/Y204^ (green) and vinculin (red). Scale bars = 20 μm. **(f)** Colocalization of pERK1/2^T202/Y204^ to vinculin was quantified using Pearson’s coefficient (*n* > 30 cells across three separate experiments). **(g–j)** Mechanocomplex formation is force and eIF6 dependent. ECs transfected with si Scr or si eIF6 were subjected to force on PECAM-1 for 0 min (no force [NF]) or 30 min (force [F]). Colocalization of FAK with actin (g), pERK^T202/Y204^ with actin (h), RACK1 with α-actinin (i), and pERK^T202/Y204^ with α-actinin (j), using Pearson’s coefficient, following force (*n* > 30 cells across three separate experiments). Values in d–j are mean ± SEM, and significance in d and f was determined by two-sided *t* test and in g–j by two-way ANOVA. *, P < 0.05; **, P < 0.01; ***, P < 0.001; ****, P < 0.0001.

The requirement for both eIF6 and localized ERK activation in force-induced focal adhesion remodeling led us to hypothesize that these two events are linked and that eIF6 is also important for localized activation of ERK1/2 in response to force. Superresolution confocal microscopy showed increased pERK^T202/Y204^ in both si Scr– and si eIF6–transfected cells subjected to force (Fig. 5, h and i), consistent with the Western blotting results presented in Fig. 5, a and b. However, when we examined the spatial activation of ERK1/2 by assaying colocalization of pERK^T202/Y204^ with vinculin, we found that while force promoted localization of pERK^T202/Y204^ at focal adhesions in control cells (Fig. 5, h and j), this force-induced colocalization was lost in eIF6-depleted cells (Fig. 5, h and j). Again, importantly, we found no differences between nonforce conditions, showing that eIF6 knockdown does not alter unstimulated pERK^T202/Y204^ levels or localization in serum-starved ECs. In addition, similar defects in the spatial localization of pERK^T202/Y204^ were observed in migrating (and thus mechanically active) eIF6-depleted cells in the absence of external force (Fig. S5, a–d). Conversely, eIF6 overexpression induced a small increase in the localization of pERK^T202/Y204^ to focal adhesions (Fig. S5, e and f). Taken together, these data demonstrate that eIF6 regulates the correct spatial mechanoactivation of ERK1/2 and is essential for the cellular response to force.

### Role of contractility and mechanocomplex formation in focal adhesion remodeling

To explore the role of contractility in the force response, we treated ECs with Rho kinase (ROCK) inhibitor Y-27632 to inhibit contractility before examining their force response. In vehicle-treated cells, application of force on PECAM-1 resulted in both increased global activation of ERK1/2 (Fig. S4 d) and increased localized ERK1/2 activation at focal adhesions (Fig. S4 e). In Y-27632–treated cells, we found that although force induced global ERK1/2 activation, in agreement with previous studies (Helfman and Pawlak, 2005), the localization of activated ERK at focal adhesions is lost (Fig. S4, d and e). Furthermore, we examined possible effects of Y-27632 on force-induced focal adhesion remodeling and found that Y-27632–treated cells did not display force-induced increases in focal adhesion area and number (Fig. S4, f and g). These results collectively show that contractility is required for force-induced localized ERK1/2 activation and downstream focal adhesion remodeling.

**Figure 6.**
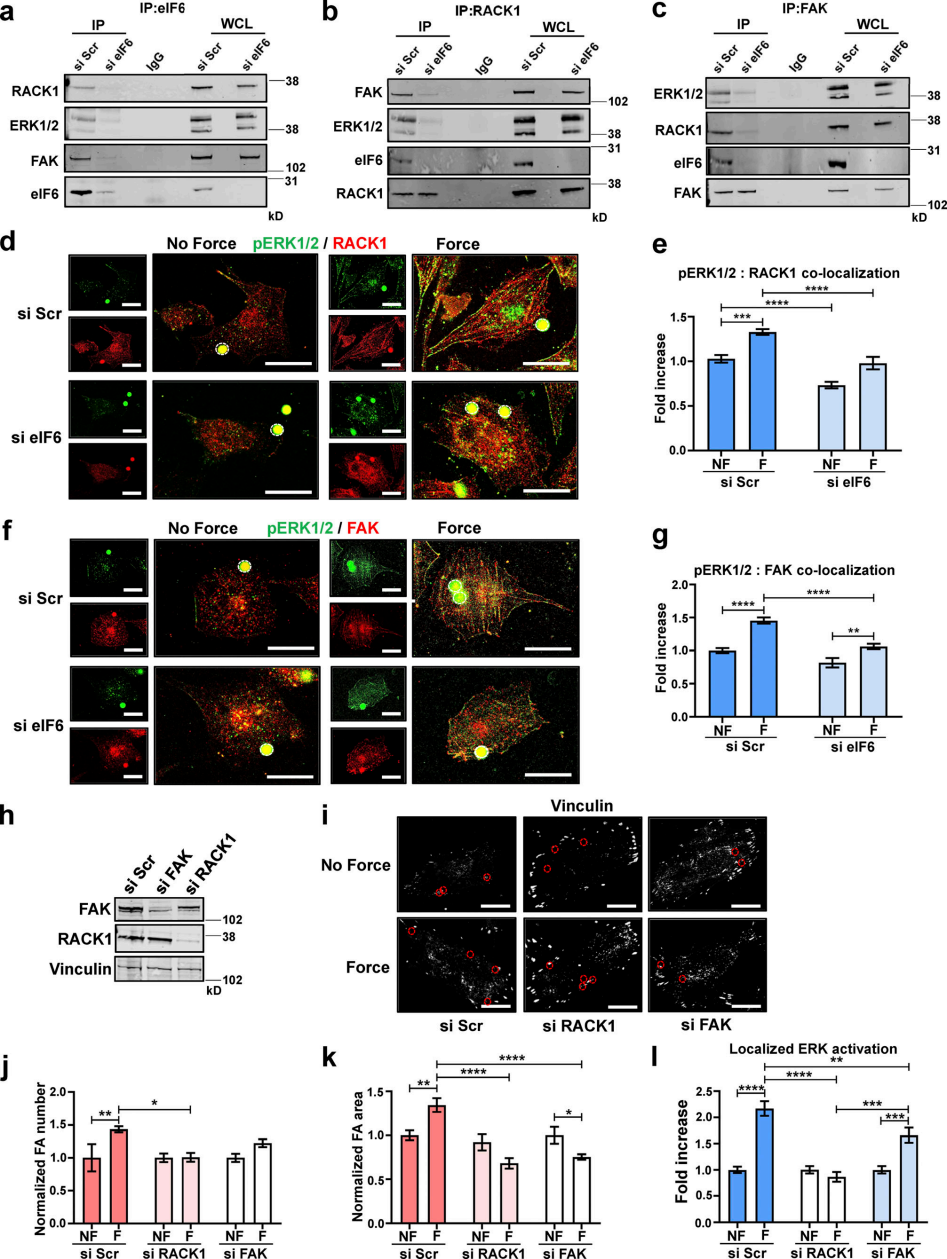
**eIF6 stabilizes mechanocomplexes.** ECs were transfected with si Scr or si eIF6. **(a)** Immunoprecipitants, using an IgG- or eIF6-specific antibody, from si Scr and si eIF6 EC lysates were tested for association of RACK1, ERK1/2, and FAK; representative blots show eIF6-dependent associations (*n* = 3). **(b)** Immunoprecipitants, using an IgG- or RACK1-specific antibody, from si Scr and si eIF6 EC lysates were tested for association of ERK1/2, FAK, and eIF6; representative blots show eIF6-dependent associations (*n* = 3). **(c)** Immunoprecipitants, using an IgG- or FAK-specific antibody, from si Scr and si eIF6 EC lysates were tested for association of RACK1, ERK1/2, and eIF6; representative blots show eIF6-dependent associations (*n* = 3). **(d–g)** si Scr or si eIF6 ECs were exposed to force for 0 min (no force [NF]) or 30 min (force [F]). **(d and f)** Representative immunofluorescent micrographs showing colocalization of pERK^T202/Y204^ (green) and RACK1 (red; d), and pERK^T202/Y204^ (green) and FAK (red; f) following application of force. Magnetic beads are highlighted by white circles. Scale bars = 20 μm. **(e and g)** Image analysis quantification of colocalization of pERK^T202/Y204^ with RACK1 (e) and pERK^T202/Y204^ with FAK (g), using Pearson’s coefficient, following force (*n* > 30 cells across three separate experiments). **(h)** ECs were transfected with si Scr, si RACK1, or si FAK. Mechanical force was applied for 0 min (NF) or 30 min (F) to si Scr, si FAK, and si RACK1 ECs. **(i)** Representative superresolution immunofluorescent micrographs showing focal adhesions (vinculin; white) in ECs following force. Magnetic beads are highlighted by red circles. Scale bars = 20 μm. **(j–l)** Quantification of mean frequency of focal adhesions per cell (j), mean area of vinculin-positive focal adhesions (k), and localization of pERK^T202/Y204^ at focal adhesions (l; *n* > 30 cells across three separate experiments). Values in e, g, and j–l are mean ± SEM, and significance was determined by two-way ANOVA. *, P < 0.05; **, P < 0.01; ***, P < 0.001; ****, P < 0.0001. FA, focal adhesion; IP, immunoprecipitation; WCL, whole-cell lysate. Source data are available for this figure: SourceData F6.

To investigate the molecular mechanisms by which eIF6 regulates the spatial activation of ERK1/2, we considered the scaffold protein RACK1 (Vomastek et al., 2007), which interacts with actin and regulates focal adhesions (Cox et al., 2003; Neasta et al., 2016). Importantly, in addition to binding ERK1/2, RACK1 is an interactor of eIF6 (Gallo and Manfrini, 2015; Miluzio et al., 2009). Indeed, using immunoprecipitation, we observed an association among eIF6, RACK1, and ERK1/2 in our system, which was not seen in si eIF6–transfected cells (Fig. 6 a). The same associations were also seen using the reverse coimmunoprecipitations, and importantly, these associations were reduced or absent in eIF6-depleted cells (Fig. 6 b). Given that FAK also associates with RACK1 and regulates pERK^T202/Y204^ localization to focal adhesions (Kiely et al., 2009; Vomastek et al., 2007), we also tested for the presence of FAK in the immunoprecipitants and found that FAK coimmunoprecipitates with eIF6, RACK1, and ERK1/2 and that loss of eIF6 disrupts these associations (Fig. 6 c). Overall, our results point toward a role for eIF6 in stabilization of an eIF6–RACK1–ERK1/2–FAK complex that is necessary for focal adhesion dynamics and force-induced structural remodeling.

To further evaluate the relevance of this complex, we assessed the subcellular localization of the mechanocomplex components in response to force. For these studies, we used confocal imaging (rather than coimmunoprecipitations), as this approach has the bonus of providing spatial information. Colocalization analyses revealed that force application induced an increase in the colocalization of pERK1/2^T202/Y204^ and RACK1 as well as pERK1/2^T202/Y204^ and FAK (Fig. 6, d–g), which occurred at actin filaments (indicated by colocalization with phalloidin) and focal adhesions (indicated by colocalization with α-actinin; Fig. S5, g–j). These increases were blocked by siRNA against eIF6, suggesting that eIF6 provides a spatial cue for the recruitment of other proteins that direct proper localization of activated ERK1/2 and subsequent cytoskeletal remodeling (Fig. 6, d–g; and Fig. S5, g–j). To investigate the importance of FAK and RACK1 specifically, we used siRNAs to knock down endogenous RACK1 and FAK (Fig. 6 h) before examining the cellular response to force. We found that following either RACK1 or FAK knockdown, ECs fail to respond to force, as they do not display force-induced focal adhesion growth (Fig. 6, i–k). We also investigated if localized ERK1/2 activation at focal adhesions was disturbed in these cells. We found that while si Scr–transfected cells showed increased localized ERK1/2 activation in response to force, this response was abolished in RACK1 knockdown cells and significantly reduced in FAK knockdown cells (Fig. 6 l). These results show that RACK1 and FAK are required for both localized activation of ERK1/2 at focal adhesions and focal adhesion remodeling in response to force. Taken together, these results suggest that eIF6 facilitates and regulates the force-induced interactions of a mechanosensitive eIF6–RACK1–ERK1/2–FAK protein complex, which localizes with the actin cytoskeleton and focal adhesions. This complex regulates force-dependent focal adhesion growth, correct spatial activation of ERK1/2, and downstream mechanical responses.

## Discussion

Growing evidence suggests that components of the protein synthesis machinery, including ribosomal proteins, tRNAs, eukaryotic initiation and elongation factors, and aminoacyl-tRNA synthetases (Brown et al., 2010; Diebel et al., 2016; Guo and Schimmel, 2013; Mateyak and Kinzy, 2010; Warner and McIntosh, 2009) can function in other cellular roles, thus challenging the tenet that the protein translation apparatus has a monolithic function. The eukaryotic initiation factor eIF6 is a component of the protein translation apparatus that binds as a chaperone to the large 60S ribosomal subunit during the final stages of ribosomal biogenesis and transport out of the nucleus. In the cytoplasm, it is involved in preventing unproductive formation of 80S monosomes in the absence of a 43S–mRNA initiation complex (Ceci et al., 2003; Weis et al., 2015). Despite its role in ribosomal subunit joining, eIF6 is not bound to the 80S ribosome during the elongation step of mRNA translation and generally not required to maintain steady-state protein synthesis levels in the cell (Brina et al., 2015a; Chendrimada et al., 2007; Clarke et al., 2017; Gandin et al., 2008). Similar behaviors have been found for the analogous 40S ribosomal chaperone protein RACK1, which is also involved in a number cell signaling pathways and, intriguingly, has been reported to form a complex with eIF6 (Ceci et al., 2003; Guo et al., 2011). We now show that unstimulated eIF6-depleted ECs exhibit unchanged nascent protein synthesis, polysome profiles, and cytoskeleton protein expression. This result is consistent with previous reports that showed normal polysome profiles in fibroblasts and HeLa cells with ∼50%–80% knockdown of eIF6 as well as in eIF6 heterozygous mice (Brina et al., 2015a; Chendrimada et al., 2007; Clarke et al., 2017; Gandin et al., 2008), although there is some discrepancy in the literature as to the effects of eIF6 loss on 60S and 80S profiles perhaps due to cell-dependent variability and differences in knockdown efficiency (Brina et al., 2011; Chendrimada et al., 2007). In ECs, even an ∼90% knockdown of eIF6 showed no visible defects in basal protein synthesis and, importantly, no changes in the key cytoskeleton components analyzed.

Our results demonstrate that eIF6 is required for the cellular response to both cell-generated and externally applied forces via regulation of mechanosensitive pathways. We show that loss of eIF6 lowers the magnitude of traction forces cells exert on their substrate, lowers their stiffness, and suppresses the ability of cells to respond to external tensional forces. An advantage of our force application protocol was the ability to examine early events, including activation of key cytoskeleton signaling mediators and focal adhesion growth. We showed that these early mechanotransduction responses do not require new proteins to be made, yet they do require eIF6, thus allowing decoupling of the translational versus signaling functions of eIF6. That is, of course, not to say that cytoskeleton-dependent functions over a longer time frame (e.g., cell migration, EC alignment) that require proteolytic cleavage and degradation of proteins over multiple cycles of focal adhesion assembly and disassembly will not be affected by inhibition of translation and eIF6 depletion. In addition, while a requirement for eIF6 for translation of specific transcripts cannot be unequivocally excluded, the presented data support and extratranslational role for eIF6. Indeed, our results, consistent with previous reports (Brina et al., 2015a; Gandin et al., 2008; Miluzio et al., 2016), show that eIF6 is required for insulin-induced nascent protein synthesis; we also show that eIF6 is not required for nascent protein synthesis in response to force. These data raise the intriguing possibility that the requirement for eIF6 in regulation of protein synthesis is context and stimulus dependent. Overall, these critical observations uncovered a novel, noncanonical role for eIF6 in the regulation of cell mechanics, independent of translation, and expand the ever-increasing repertoire of the extratranslational functions found for members of the protein synthesis apparatus.

RACK1 is a scaffold protein that has been shown to be critical for shuttling or anchoring several proteins to specific subcellular locations and involved in a variety of cell signaling pathways, including the regulation of focal adhesions (Cox et al., 2003; Liliental and Chang, 1998; Nilsson et al., 2004; Vomastek et al., 2007). Importantly, RACK1 also plays a key role in the translation machinery, as it binds to the 40S ribosomal subunit (Adams et al., 2011; Nilsson et al., 2004). RACK1 has also been described to exist in a complex with eIF6 across diverse species (Ceci et al., 2003; Guo et al., 2011); however, the functional significance of this interaction is poorly understood. We now establish an eIF6-dependent RACK1–ERK1/2–FAK complex that is responsible for the distinct, spatial localization of activated ERK1/2 at focal adhesions. We show that while eIF6 is not required for the global mechanical activation of ERK1/2 in response to tensional force, it is essential for the localized activation of ERK1/2 at focal adhesions both under basal conditions and in response to mechanical force. Tension developed in focal adhesions and actin stress fibers has been proposed to be a critical mechanism driving ERK signaling, as spatially distinct pERK localization on these structures has been described (Hirata et al., 2015). We also show that localized ERK1/2 activation at focal adhesions in response to force is required for downstream focal adhesion remodeling. Furthermore, both RACK1 and FAK have been shown to regulate ERK activation at focal adhesions and downstream focal adhesion remodeling (Vomastek et al., 2007). The relationship of this mechanocomplex with the ribosome is currently unknown. Unlike most ribosome-associated proteins, RACK1 and eIF6 are not members of actively translating ribosomes but, rather, play analogous roles in preventing unproductive 80S monosome formation in the absence of translation-competent mRNAs. Both proteins not only lie at the nexus between the cell signaling and translational machineries but also may act as signaling hubs away from the ribosome. By using a ROCK inhibitor, Y-27632, we have demonstrated that contractility is important for focal adhesion remodeling in response to force and, indeed, that eIF6-depleted ECs phenocopy ROCK-inhibited ECs. When considered with our finding that eIF6 forms an ERK–RACK1–FAK mechanocomplex, our model adds to previously published work that suggests that contractility and activated ERK localization are linked (Lavoie et al., 2020; Tanimura and Takeda, 2017). In a force-dependent context, regulation of active ERK is important both upstream of contractility pathways, in initiation of cell contractility, and downstream, where spatial regulation of ERK is required for focal adhesion remodeling (Lavoie et al., 2020; Tanimura and Takeda, 2017).

In summary, our results show that formation of an eIF6-dependent mechanocomplex is required to regulate the structural components of the cell and dynamic remodeling in response to mechanical stimulation by mediating localization of pERK^T202/Y204^ to focal adhesions. Hence, our findings provide a novel physical and signaling link between the cytoskeletal and translational machinery that is required for dynamic remodeling of the cytoskeleton in response to mechanical stimulation by coordinating correct localization of cellular signaling cascades.

## Materials and methods

### Cell culture and transfections

Bovine aortic ECs, human embryonic kidney 293A cells, and human epidermoid carcinoma A431 cells were cultured in DMEM (Corning) supplemented with 10% FBS and 1% penicillin and streptomycin (Gibco). Human umbilical vein ECs and mouse ECs were cultured in EGM2 growth medium (Lonza). Cells were cultured at 37°C in a humid atmosphere of 5% CO_2_. siRNA reverse transfections for Scr (siGENOME, D-001810-10; Dharmacon), eIF6 (SMARTpool, L-010096-00; Dharmacon) eIF6 1 (ON-TARGETplus, J-010096-07; Dharmacon), eIF6 2 (ON-TARGETplus, J-010096-08; Dharmacon), RPL7 (SMARTpool, L-013727-00; Dharmacon), RACK1 (SMARTpool, L-006876-00; Dharmacon), FAK (SMARTpool, L-003164-00; Dharmacon), ERK1 (SMARTpool, L-003592-00; Dharmacon), or ERK2 (SMARTpool, L-003555-00; Dharmacon) were performed using the Lipofectamine RNAiMAX reagent (Invitrogen).

Plasmids included pCMV-myc-ERK2-MEK1_fusion (Addgene plasmid #39194; http://n2t.net/addgene:39194; Research Resource Identifier [RRID]: Addgene_39194; Robinson et al., 1998), pFLAG-CVM-hERK1 (Addgene plasmid #49328; http://n2t.net/addgene:49328; RRID: Addgene 49328), and pCMV5-myc-ERK2 (Addgene plasmid #39222; http://n2t.net/addgene:39222; RRID: Addgene_39222; Xu et al., 2001).

### Cloning and adenoviral generation

WT eIF6 was cloned into the pENTR/TOPO entry vector of the Gateway System (Invitrogen) using the KOD Hot Start high-fidelity polymerase. After confirmation of successful cloning by Sanger sequencing, the constructs were subcloned into the pAd/CMV/V5-Dest destination vector by LR Clonase II reaction. All steps were performed according to the manufacturer’s instructions. The destination vector encoding eIF6 or LACZ was linearized by PacI digestion and transfected into human embryonic kidney 293A cells for adenoviral generation and subsequent amplification according to the manufacturer’s instructions.

### Antibodies, inhibitors, and other reagents

The antibodies included total (t)-eIF6 (611120; BD Biosciences), t-paxillin (610568; BD Biosciences), t-actin (Ab179467; Abcam), α-actinin (ab50599; Abcam), t-eIF6 (D16E9; Cell Signaling Technology), t-ERK1/2 (9102; Cell Signaling Technology), t-FAK (3285; Cell Signaling Technology), PECAM-1 (a gift from Peter Newman, Medical College of Wisconsin, Milwaukee, WI; Collins et al., 2012), phospho (p)-p-ERK1/2^T202:Y204^ (9106; Cell Signaling Technology), t-RPL7a (2415; Cell Signaling Technology), t-RPL26 (2065; Cell Signaling Technology), p-Akt^S473^ (4060; Cell Signaling Technology), t-Akt (9272; Cell Signaling Technology), p-p70s6K^T389^ (9205; Cell Signaling Technology), p70s6K (9202; Cell Signaling Technology), p-mTOR^S2448^ (5536; Cell Signaling Technology), t-mTOR (4517; Cell Signaling Technology), α-actinin (ab50599; Abcam), t-cofilin (ab54532; Abcam), t-RPL10a (WH0004736M1; Sigma-Aldrich), t-RPL23 (SAB4503628; Sigma-Aldrich), t-vinculin (V9131; Sigma-Aldrich), p-FAK^Y397^ (44-624; Thermo Fisher Scientific), puromycin (MABE343; EMD Millipore), RACK1 (sc-17754; Santa Cruz Biotechnology), Alexa Fluor 488 goat anti-mouse (A11001; Invitrogen), Alexa Fluor 488 goat anti-rabbit (A11034; Invitrogen), Alexa Fluor 568 goat anti-mouse (A11061; Invitrogen), Alexa Fluor 568 goat anti-rabbit (A11011; Invitrogen), Alexa Fluor 647 goat anti-mouse (A21235; Invitrogen), Alexa Fluor 647 goat anti-rabbit (A21244; Invitrogen), Alexa Fluor 647 goat anti-rat (A21247; Invitrogen), Alexa Fluor 790 goat anti-rabbit (A11367; Invitrogen), Alexa Fluor 680 goat anti-rabbit (A21076; Invitrogen), Alexa Fluor 790 goat anti-mouse (A11375; Invitrogen), and Alexa Fluor 680 goat anti-mouse (A21058; Invitrogen).

Inhibitors included cycloheximide (UltraPure; VWR), RNase Block Ribonuclease Inhibitor (Agilent), puromycin (Sigma-Aldrich), emetine (Sigma-Aldrich), and Y-27632 (EMD Millipore).

### Immunofluorescence staining

Cells were permeabilized in 0.2% Triton X-100 (Sigma-Aldrich) and blocked with 10% normal goat serum/1% BSA. Cells were incubated with primary antibodies (1:100) before incubation with Alexa Fluor 488-, 568-, and/or 647-conjugated secondary antibodies (1:150; Invitrogen) followed by Alexa Fluor 488-, 568-, and/or 647-conjugated phalloidin (Invitrogen) and DAPI (Invitrogen) and mounted with SlowFade Gold (Invitrogen).

### Confocal and superresolution microscopy

Images were acquired on a Zeiss LSM 880 upright confocal microscope using a Zeiss Plan-Apochromat 20×/1 NA air objective or a Zeiss Plan-Apochromat 63×/1.4 NA oil objective equipped with 405-nm solid-state, 488-nm argon, and 561-nm diode lasers and main beam splitter fluorescent filters. For confocal images, a Quasar detector was used. For superresolution images, airyscan mode was used with an airyscan detector. Images were acquired at room temperature using Zen 2.3 software; airy processing was also performed on this software with a correction of 6.

### Coimmunoprecipitation and Western blotting

Cells were collected in lysis buffer (1 mM EGTA, 1 mM EDTA, 10 mM Tris [pH 7.4], 150 mM sodium chloride, 1% deoxycholate, 1% NP-40, and supplemented with protease and phosphatase inhibitor cocktail tablets [cOmplete mini EDTA-free and PhosStop; Roche]). Lysates were precleared with 10 μl of protein A/G PLUS Sepharose Beads (Santa Cruz Biotechnology) for 1 h at 4°C. The precleared lysates were then incubated with 20 μl of protein A/G PLUS Sepharose Beads, which had previously been coupled with the appropriate primary antibody for 2 h at 4°C on an orbital shaker. The beads were washed three times with lysis buffer supplemented with protease and phosphatase inhibitors. The immunoprecipitation complexes were eluted from the beads by boiling in 2× SDS buffer for 5 min.

For Western blotting analyses, protein lysates/coimmunoprecipitation complexes were resolved on a 4%–12% gradient gel (Invitrogen) and blotted on a nitrocellulose membrane with the appropriate primary antibodies (1:1,000) and IRDye-conjugated anti-mouse, anti-goat, or anti-rabbit secondary antibodies or the Quick Western Kit (926-69100; LI-COR), as appropriate. Images were acquired on a LI-COR Odyssey infrared scanner. Densitometric quantification of bands was performed using Image Studio software (LI-COR).

### AFM

AFM indentation was conducted on a JPK NanoWizard-1 (JPK Instruments) operating in force spectroscopy mode, mounted on an inverted optical microscope (IX-81; Olympus). AFM pyramidal cantilevers (MLCT; Bruker) with a spring constant of 0.07 N/m were used with a 35 μm glass bead attached to the cantilever tip. Before measurements with the adapted cantilevers, their sensitivity was calculated by measuring the slope of the force–distance curve in the AFM software on an empty region of the Petri dish. For cell indentation tests, the cantilever was aligned over regions in the middle of the cells using an IX-81 inverted optical microscope. For each group, 30 individual cells were tested. Force–curve acquisition was performed with an approach speed of 5 μm/s and a maximum set force of 1.5 nN. Elastic moduli were calculated from the force–distance curves by fitting the contact region of the approach curve with the Hertz contact model using the AFM software (JPK Instruments).

### Micropillar video microscopy and traction force measurements

Elastic micropillars were fabricated in polydimethylsiloxane according to a previous protocol (Chronopoulos et al., 2016). Pillar arrays were coated with human plasma fibronectin (10 μg/ml; Sigma-Aldrich) and incubated at 37°C for 1 h before measurements. Cells were plated onto the pillar substrates, left for 1 h, and then imaged for a maximum of 30 min. Time-lapse imaging of the pillars was conducted with an inverted microscope (Eclipse Ti; Nikon) operating in bright field mode at an ambient temperature of 37°C. Image sequences were recorded with a scientific complementary metal-oxide semiconductor camera (Neo sCMOS; Andor) at 0.5 Hz using a 40×/0.6 NA air objective (Nikon) over the early spreading phase (*t *< 60 min) and late-spreading phase (90 min < *t *< 120 min). The position of each pillar in the time-lapse videos was tracked using a custom MATLAB program to track the center of a point spread function of the intensity of the pillars across all frames. By selecting a location free of cells, tracking of a small set of pillars allowed a measurement of the stage drift to be obtained and corrected for in the dataset. The time-dependent displacement of a given pillar was obtained by subtracting the initial position of the pillar (zero force) from the position in a given frame. Traction forces were obtained by multiplying the pillar displacements by the pillar stiffness; the maxima for each pillar were found to obtain the peak forces across the cell.

### Nascent protein synthesis assay

Nascent protein synthesis was analyzed using nonradioactive metabolic labeling (Click-iT OPP Alexa Fluor 568 Protein Synthesis Assay Kit, C10457; Thermo Fisher Scientific) according to the manufacturer’s instructions. Briefly, cells were grown in media containing OPP reagent for 30 min. Cells were fixed (3.7% formaldehyde in PBS) and permeabilized (0.5% Triton X-100) before OPP detection. The signal intensity of incorporated OPP Alexa Fluor 568 was measured by microscopy.

As a negative control, cells were pretreated with cycloheximide (100 μg/ml) for 10 min before performing the experiment in the presence of cycloheximide (100 μg/ml). For insulin-treated cells, insulin (Sigma-Aldrich) was added at a concentration of 1 μM for 30 min.

### Polysome profiling

Growth media was supplemented with cycloheximide (100 μg/ml) for 5 min, and cells were scraped in ice-cold PBS containing cycloheximide (100 μg/ml). Cells were collected by centrifugation and lysed by vortexing for 10 s in 200 μl of polysome extraction buffer (5 mM Tris-HCl, 2.5 mM MgCl_2_, 1.5 mM KCl, 1 mM DTT, 100 μg/ml cycloheximide, 100 U/ml RNase block, 1% Triton X-100, 1% sodium deoxycholate, cOmplete, Mini, EDTA-Free Protease Inhibitor). Lysates were centrifuged at 16,000 x*g*, and supernatants collected.

Supernatants were layered on 13 ml of 5%–45% (wt/wt) sucrose density gradients (containing 20 mM Hepes [pH 7.6], 100 mM KCl, 5 mM MgCl_2_, 1 mM DTT, 100 μg/ml cycloheximide, 10 U/ml RNase block, cOmplete EDTA-Free Protease Inhibitor), then centrifuged at 40,000 rpm for 2 h at 4°C in a Beckman SW41Ti rotor. The samples were fractionated into 10 samples (each ∼1,300 μl) using density gradient fractionation (Brandel) with continuous measurement of absorbance at 254 nm.

### RNA extraction and qPCR

Total RNA extraction was performed on human umbilical vein ECs using the RNeasy Plus Mini Kit (QIAGEN), with an additional genomic DNA wipeout treatment. First-strand cDNA synthesis was performed using the Superscript III cDNA Synthesis Kit. Real-time qPCR was performed in triplicate with SYBR green and CFX96 Real-Time PCR Detection System. Thermocycling conditions were 95°C for 5 min followed by 40 cycles of 95°C for 30 s and 60°C for 1 min. Gene expression was normalized to the constitutively expressed housekeeping gene *GAPDH*, and relative expression was calculated and plotted using the ΔΔCt method. Primer sequences used were as follows: human 28S rRNA, 5′-AGT​CGG​GTT​GCT​TGG​GAA​TGC-3′, 5′-CCC​TTA​CGG​TAC​TTG​TTG​ACT-3′; human 5.8S, rRNA-5′-ACTCTTAGCGGTGGATCACTC-3′, 5′-AAG​CGA​CGC​TCA​GAC​AGG-3′; human 18S rRNA, 5′-AGG​AAT​TGA​CGG​AAG​GGC​ACC​A-3′, 5′-GTG​CAG​CCC​CGG​ACA​TCT​AAG-3′; human 45S rRNA, 5′-GTT​CGA​GGC​GGT​TTG​AGT​GA-3′, 5′-CTC​CGA​AGT​CAA​CCC​ACA​CA-3′; and human eIF6, 5′-TGG​TGC​ATC​CCA​AGA​CTT​CAA​T-3′, 5′-TCA​CAG​TCC​CCG​CCA​CA-3′.

### Bead pulling

Tosylactivated paramagnetic beads (4.5 μm) were washed with PBS and coated with an antibody to the extracellular domain of PECAM-1 (a gift from Peter Newman) or fibronectin. Prior to use, beads were quenched in 0.2 M Tris (pH 7.4) to eliminate any remaining tosyl groups. ECs were seeded on fibronectin-coated coverslips and then incubated in reduced serum media (0.5%) overnight. ECs were incubated with the beads (and inhibitors, if appropriate) for 30 min before force application and then for 5 or 30 min of force at 37°C.

To measure nascent protein synthesis during this assay, ECs were incubated with beads, puromycin (91 μM), and emetine (208 μM) before and during force application. Following force application, cells were washed two times in PBS containing cycloheximide (355 μM) before fixation in 2% formaldehyde.

### Image analysis

Image analyses were performed using ImageJ software (Schneider et al., 2012). Quantification of the colocalization was performed using the coloc2 plugin and cell area; focal adhesion number and focal adhesion area were measured using an in-house macro. For mechanically stimulated cells, only cells with one to four beads bound were used for analysis.

### Statistics

Statistical analyses were performed using GraphPad Prizm 8 software (GraphPad Software). Comparisons between groups were assessed by two-sided *t* test or two-way ANOVA with a Tukey multiple comparisons post hoc test, where appropriate. Details for statistical tests for each experiment are available in the figure legends. Data distribution was assumed to be normal, but this was not formally tested. Differences were considered significant when P < 0.05.

### Online supplemental material

Fig. S1 shows the contribution of eIF6 and ribosomal proteins to nascent protein synthesis. Fig. S2 shows that eIF6 regulates focal adhesions. Fig. S3 shows that depletion of eIF6 does not affect cytoskeletal or focal adhesion protein levels. Fig. S4 shows that tensional force on PECAM-1 elicits global focal adhesion growth that is dependent on cellular contractility. Fig. S5 shows that eIF6 regulates localization of activated ERK1/2 and mechanocomplex formation at focal adhesions.

## Supplementary Material

SourceData F1 contains original blots for Fig. 1.

SourceData F3 contains original blots for Fig. 3.

SourceData F4 contains original blots for Fig. 4.

SourceData F5 contains original blots for Fig. 5.

SourceData F6 contains original blots for Fig. 6.

SourceData FS1 contains original blots for Fig. S1.

SourceData FS2 contains original blots for Fig. S2.

SourceData FS3 contains original blots for Fig. S3.
