# Supplementary figures and images for: Eukaryotic initiation factor 6 regulates mechanical responses in endothelial cells
Source: J Cell Biol. 2022 Jan 13;221(2):e202005213. doi: 10.1083/jcb.202005213 (PMC8763864; doi:10.1083/jcb.202005213)

**eIF6**

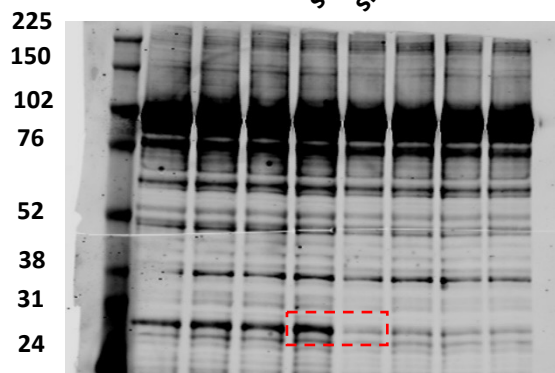

**GAPDH**

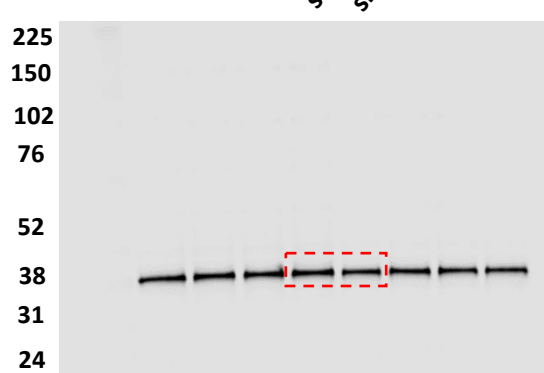

**RPL7a**

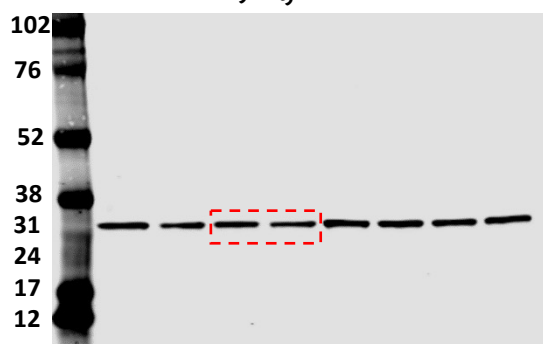

**RPL10**

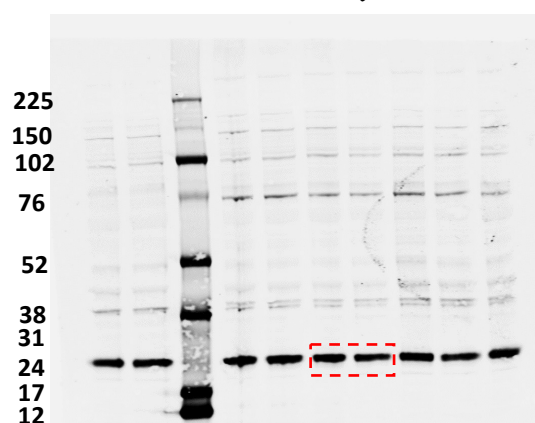

**RPL26**

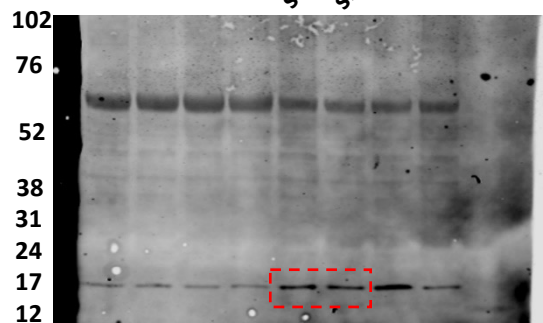

**RPL23**

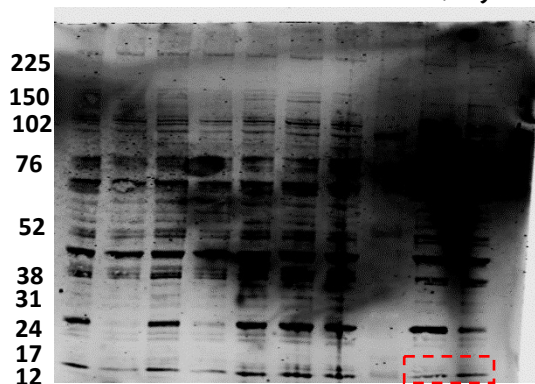

**GAPDH**

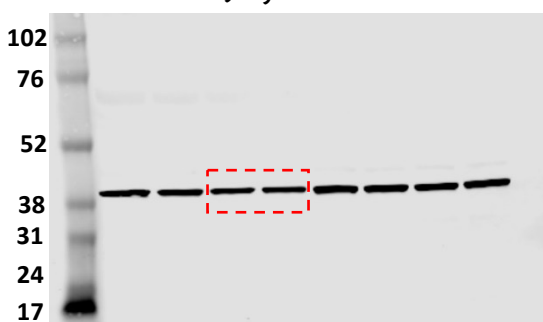

\* One GAPDH blot shown for representation

Supplement: SourceData F1 — contains original blots for Fig. 1. [file JCB_202005213_SourceDataF1.pdf]

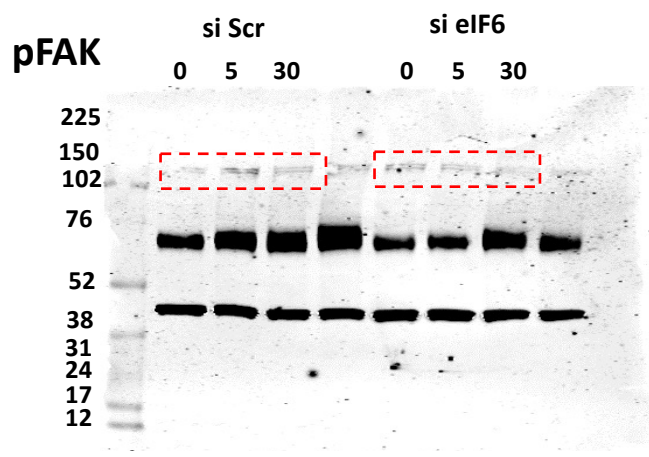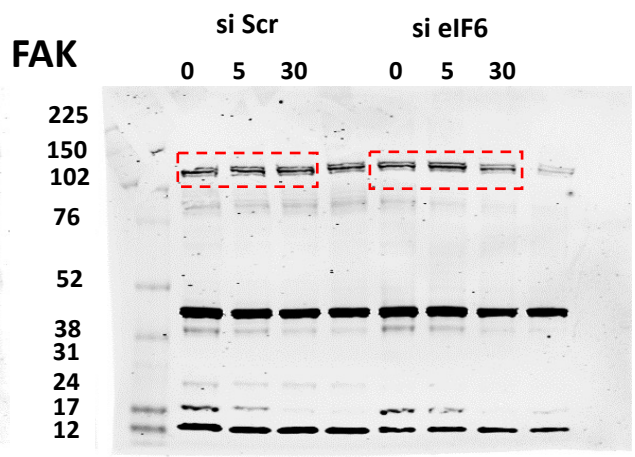

Supplement: SourceData F3 — contains original blots for Fig. 3. [file JCB_202005213_SourceDataF3.pdf]

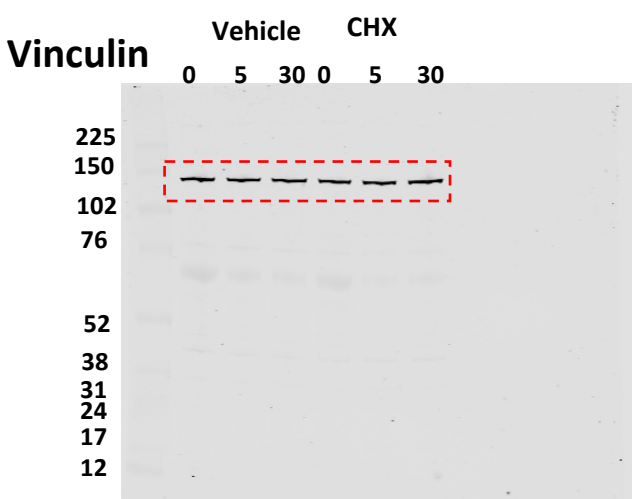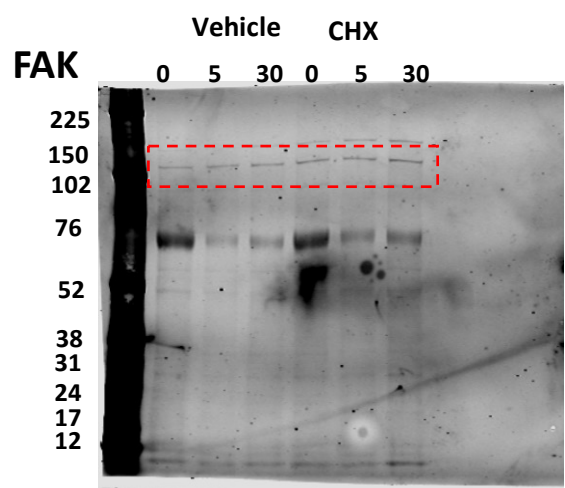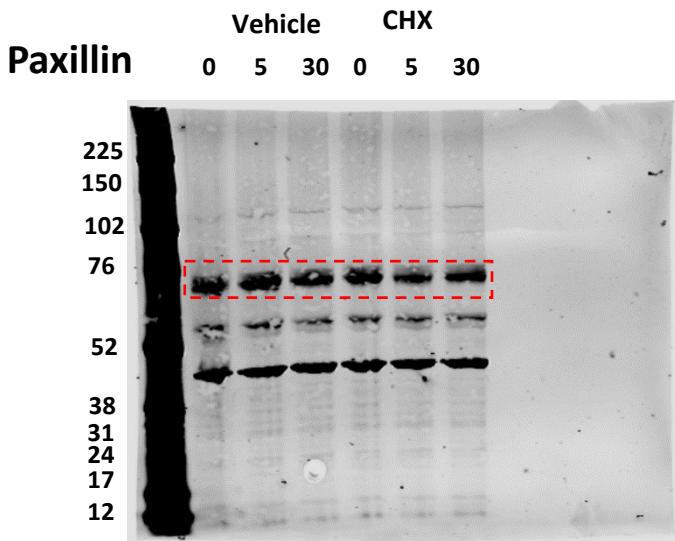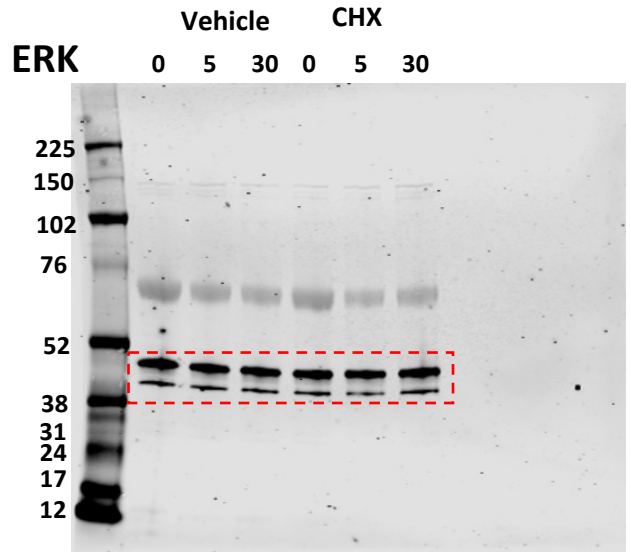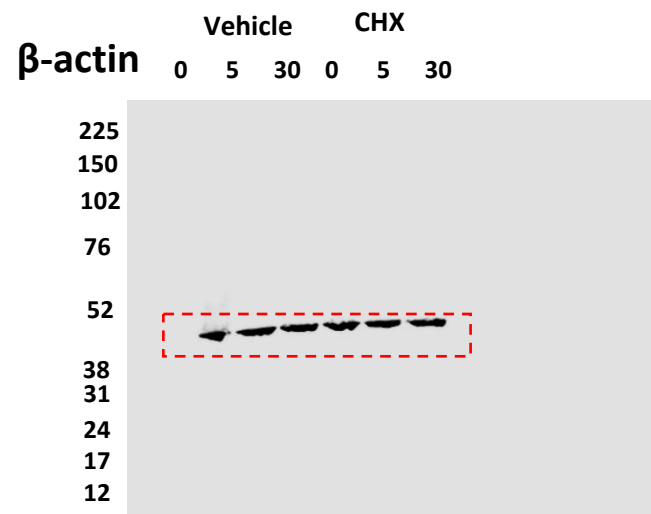

Supplement: SourceData F4 — contains original blots for Fig. 4. [file JCB_202005213_SourceDataF4.pdf]

pERK

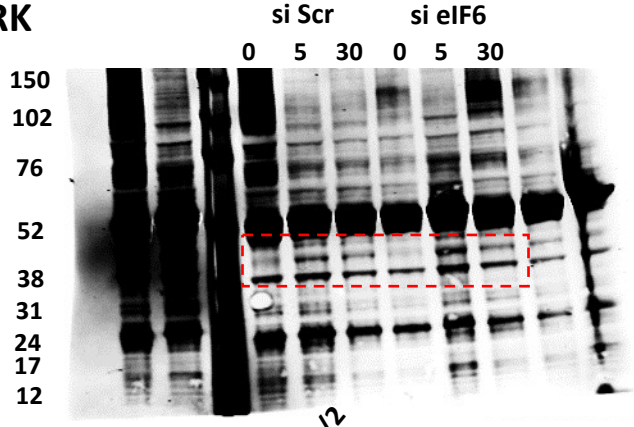

ERK

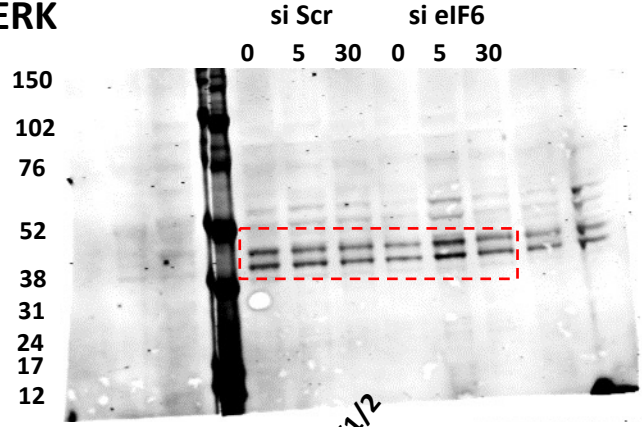

ERK1/2

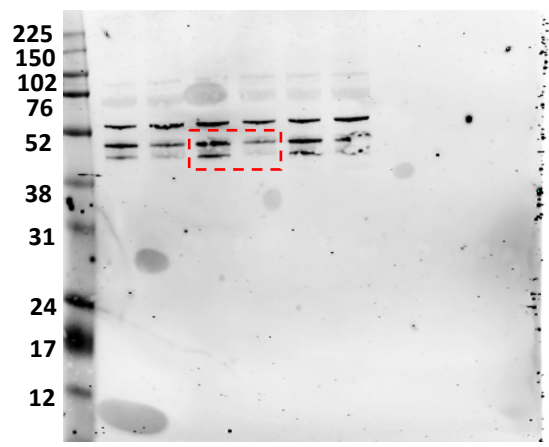

$\alpha$ -tubulin

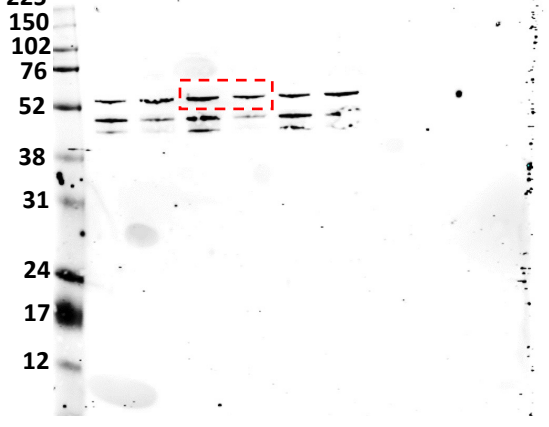

CA-ERK

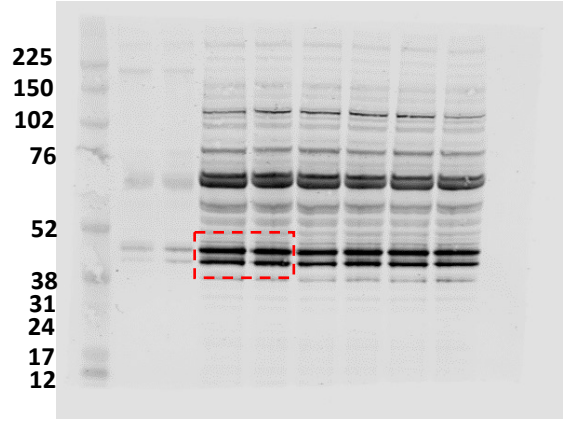

Myc tag

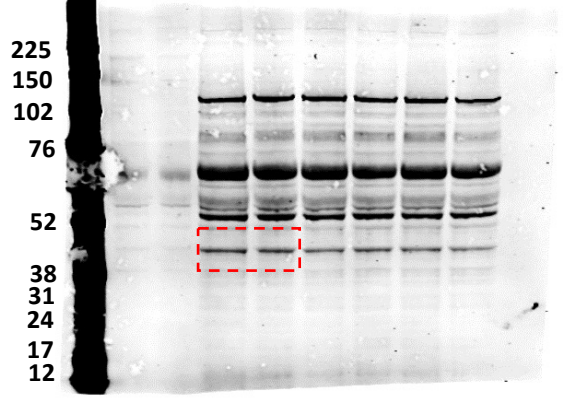

$\alpha$ -tubulin

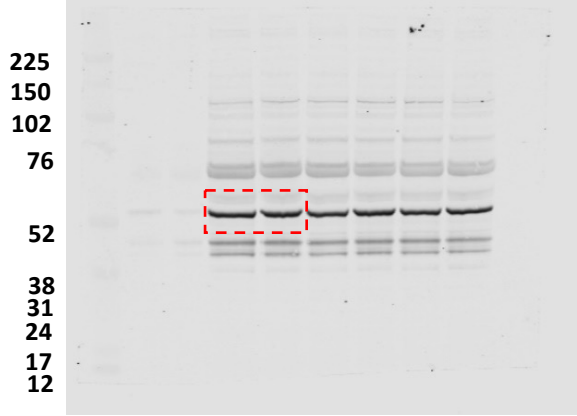

Supplement: SourceData F5 — contains original blots for Fig. 5. [file JCB_202005213_SourceDataF5.pdf]

# IP: eIF6

## RACK1

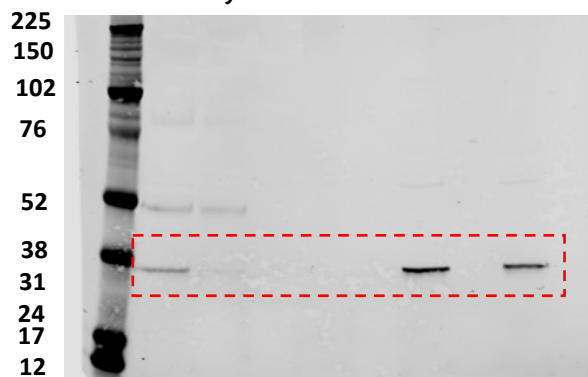

## ERK1/2

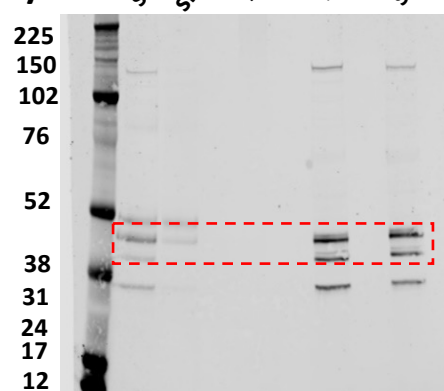

## FAK

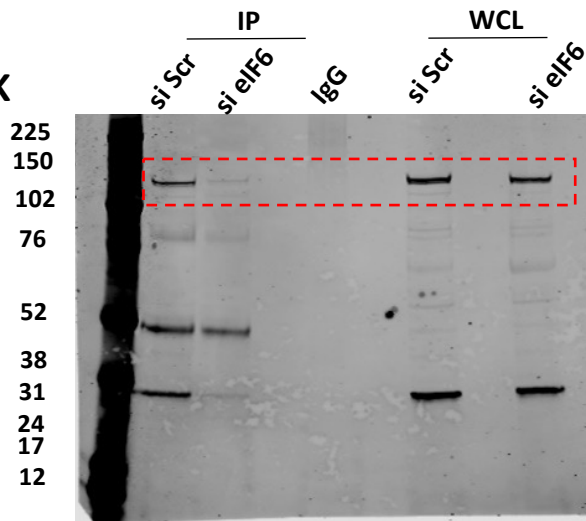

## eIF6

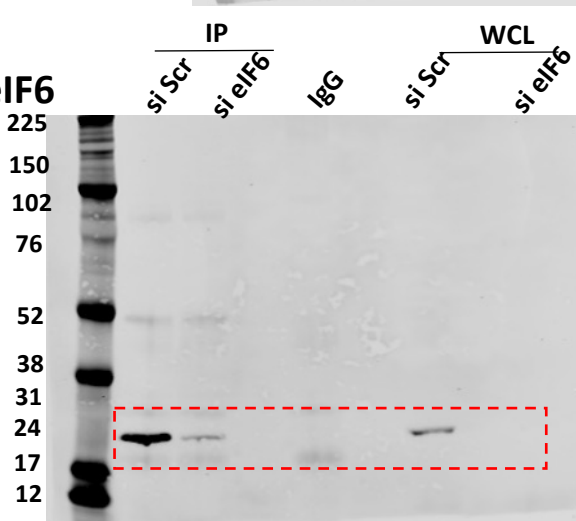

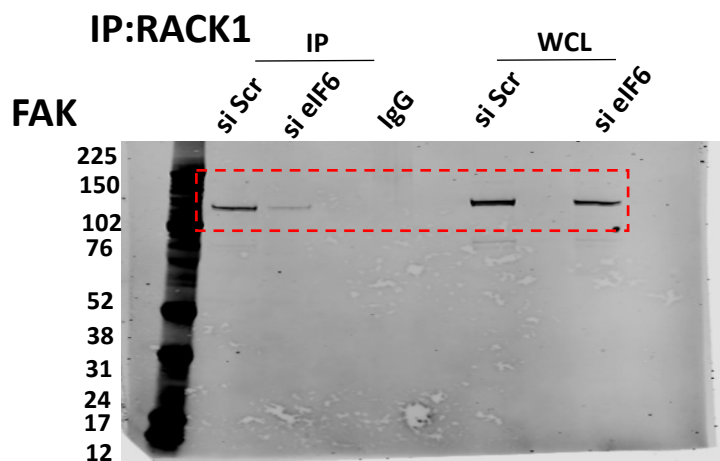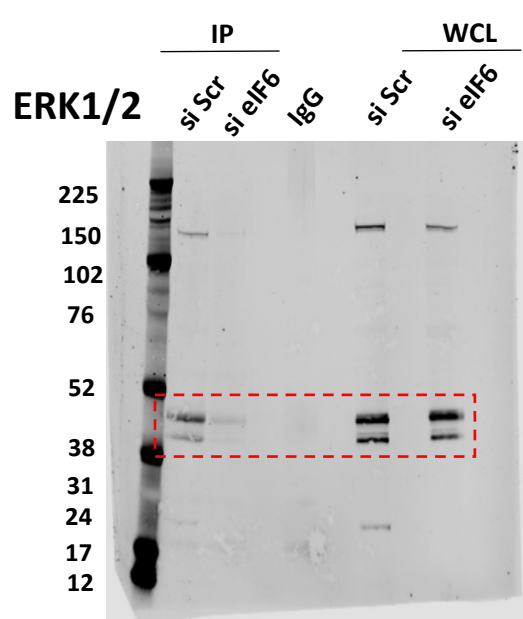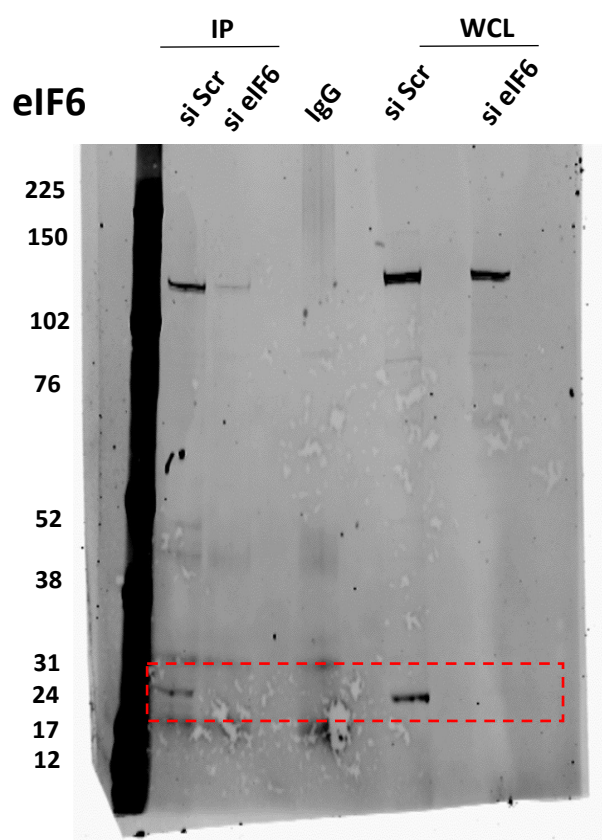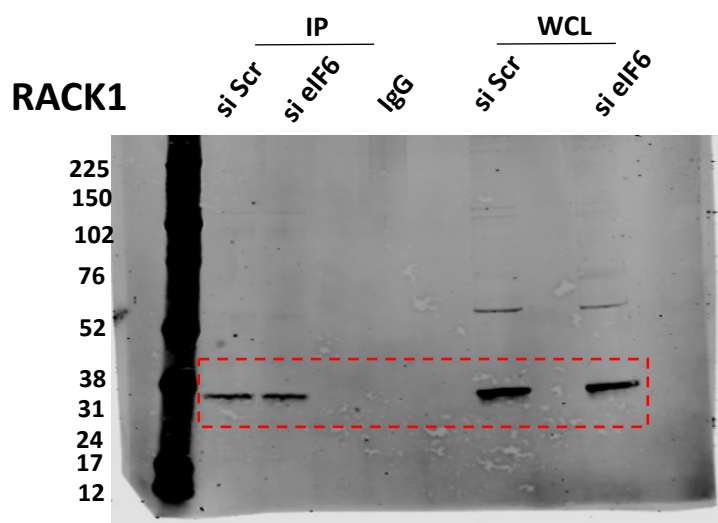

# IP:FAK

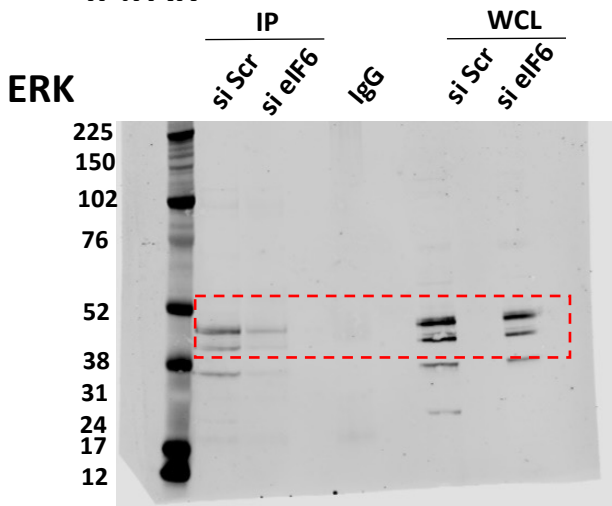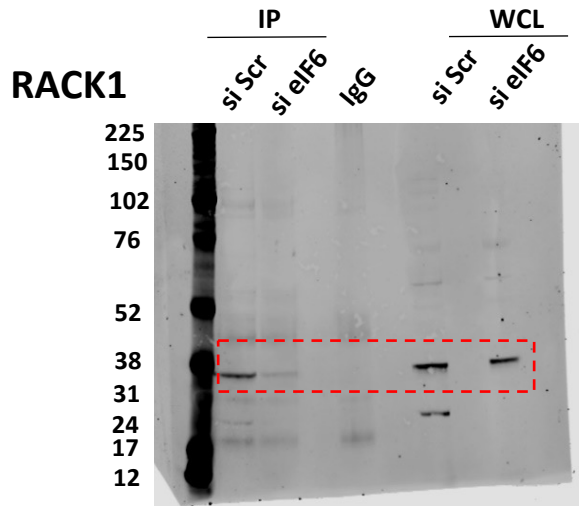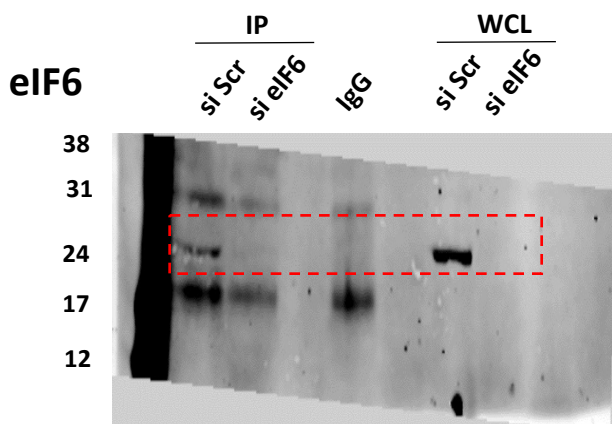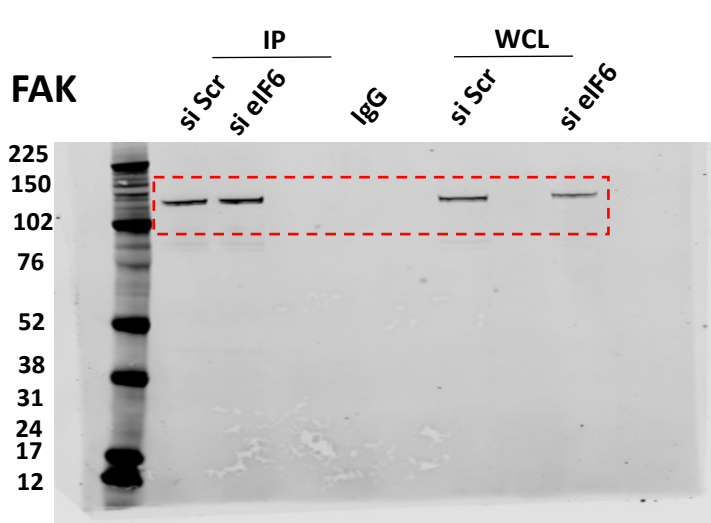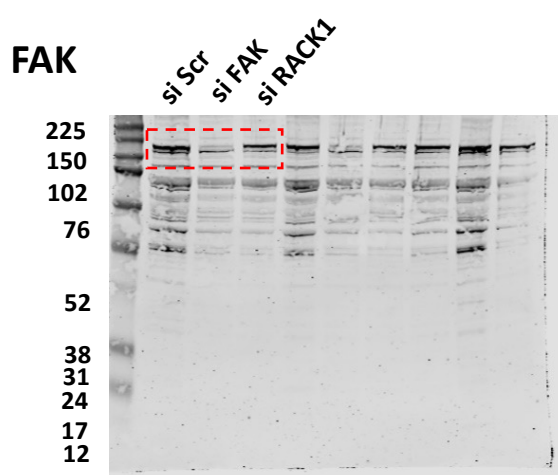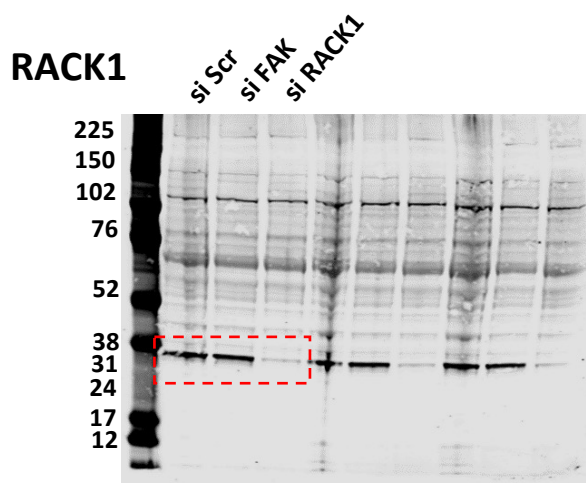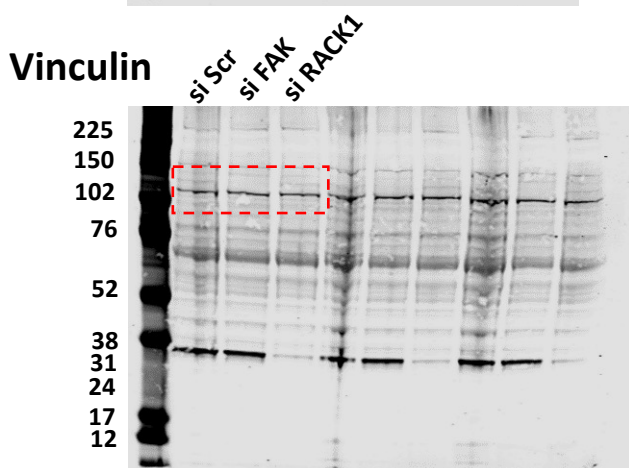

Supplement: SourceData F6 — contains original blots for Fig. 6. [file JCB_202005213_SourceDataF6.pdf]

**RPL7**

si Scr  
si RPL7

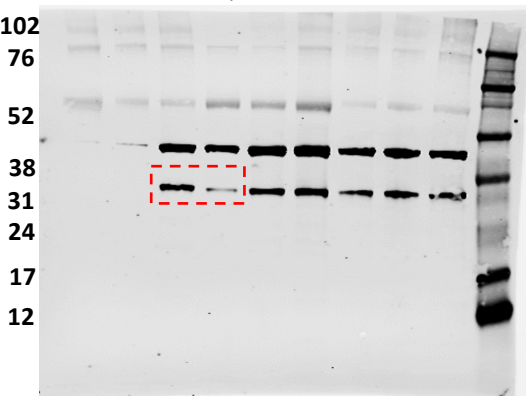

**Actin**

si Scr  
si RPL7

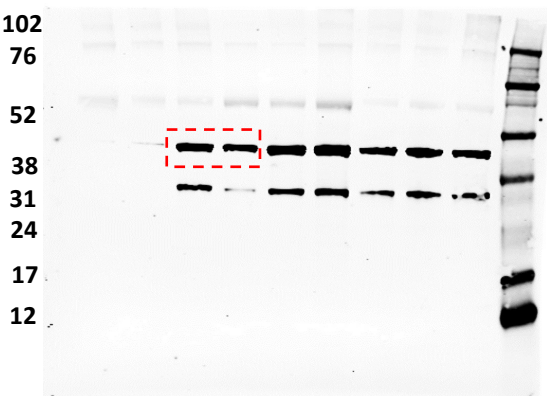

Supplement: SourceData FS1 — contains original blots for Fig. S1. [file JCB_202005213_SourceDataFS1.pdf]

eIF6 siRNA 1 + 2

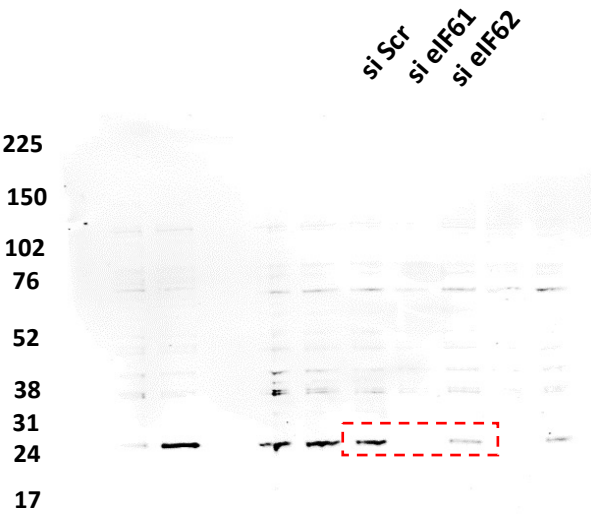

Vinculin

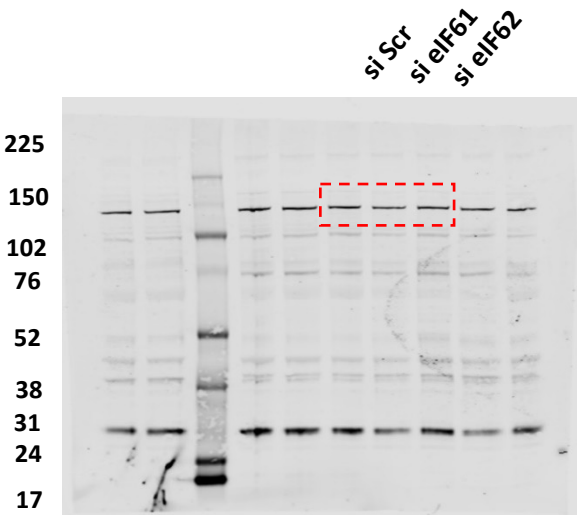

Actin

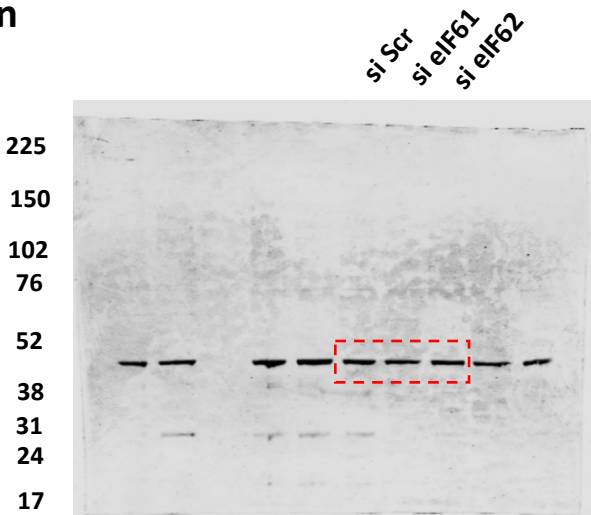

eIF6 OE

eIF6

Ad.LACZ  
Ad. eIF6

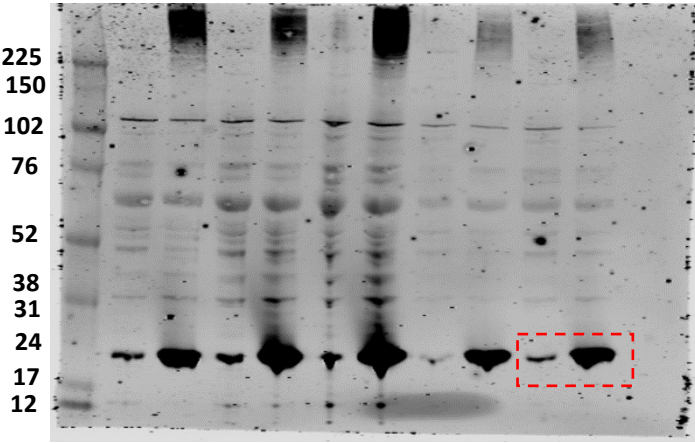

Vinculin

Ad.LACZ  
Ad. eIF6

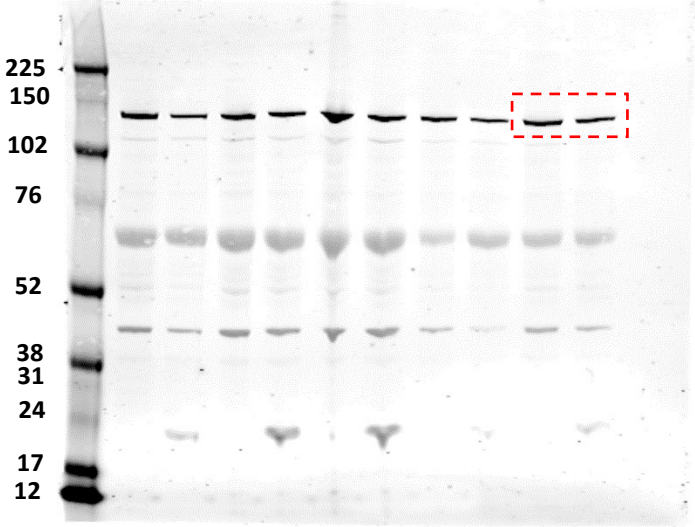

Actin

Ad.LACZ  
Ad. eIF6

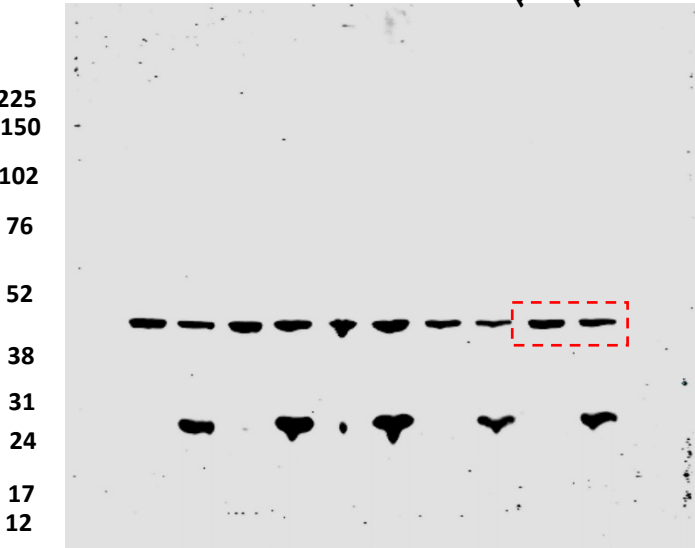

Supplement: SourceData FS2 — contains original blots for Fig. S2. [file JCB_202005213_SourceDataFS2.pdf]
