# Supplementary material for: Eukaryotic initiation factor 6 regulates mechanical responses in endothelial cells
Source: J Cell Biol. 2022 Jan 13;221(2):e202005213. doi: 10.1083/jcb.202005213 (PMC8763864; doi:10.1083/jcb.202005213)
Supplement: SourceData FS3 — contains original blots for Fig. S3. [file JCB_202005213_SourceDataFS3.pdf]

Paxillin

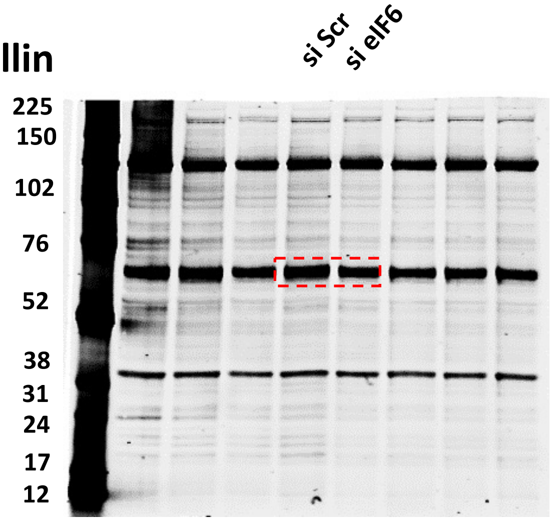

FAK

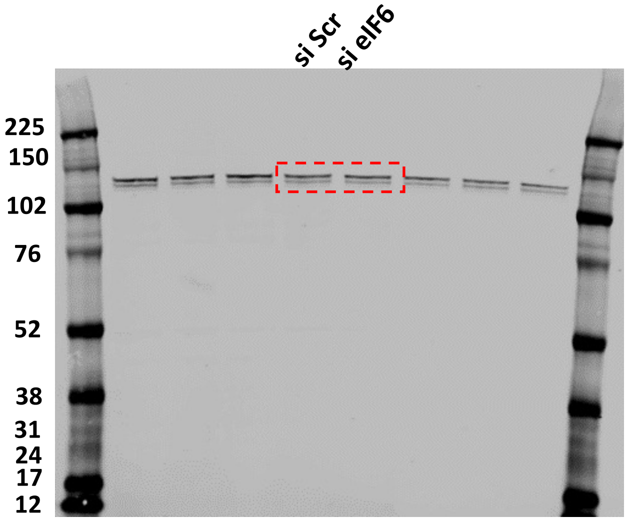

Cofilin

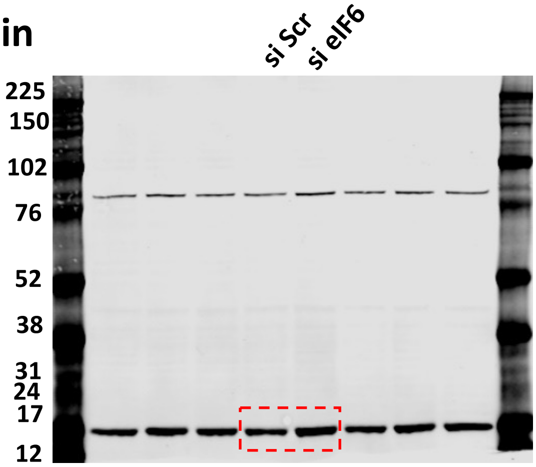

ERK1/2

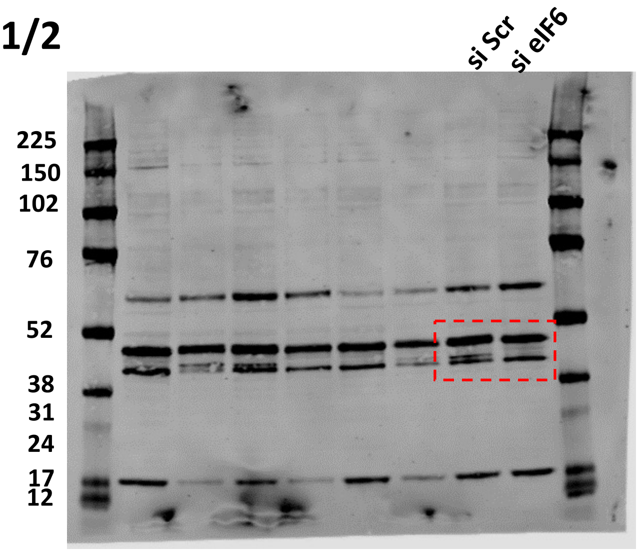

Vinculin

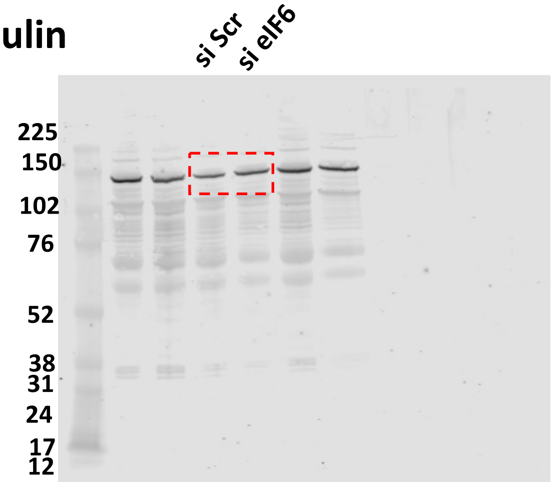

MLC

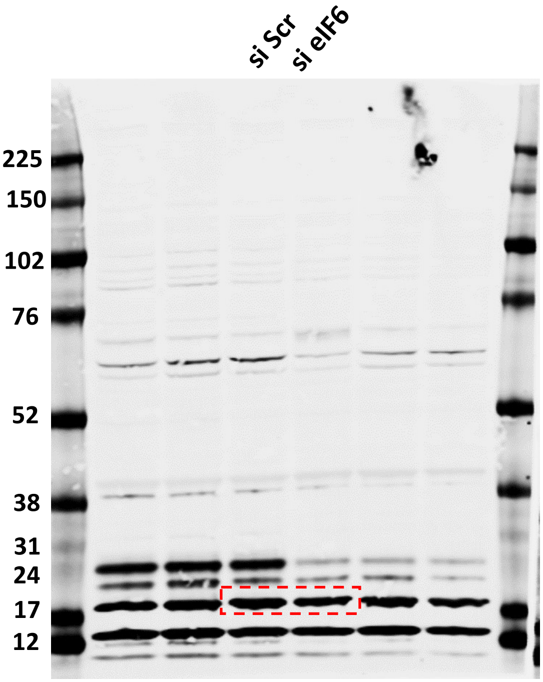

Cas

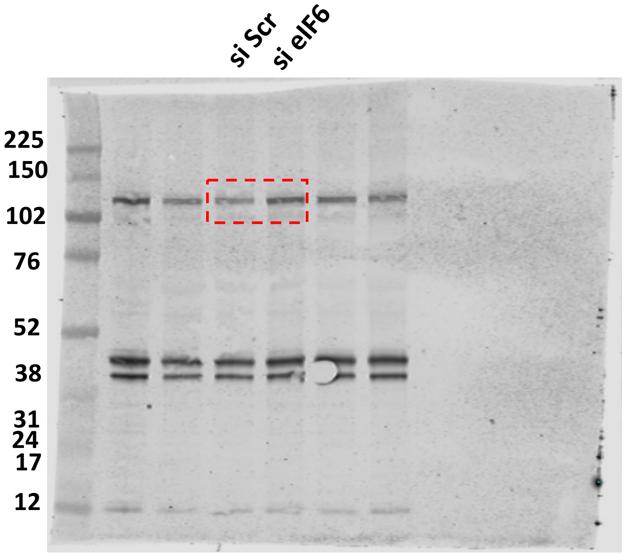

Vimentin

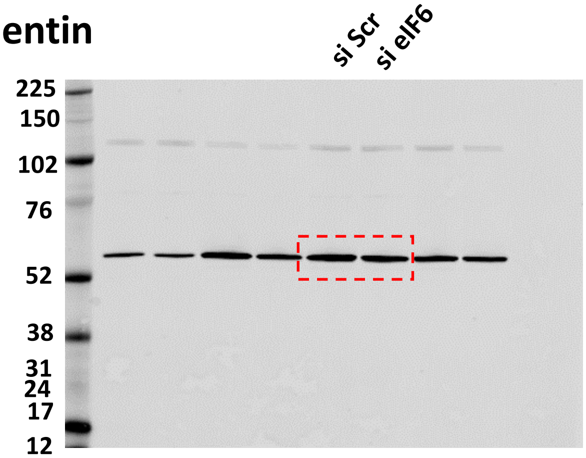

Actin

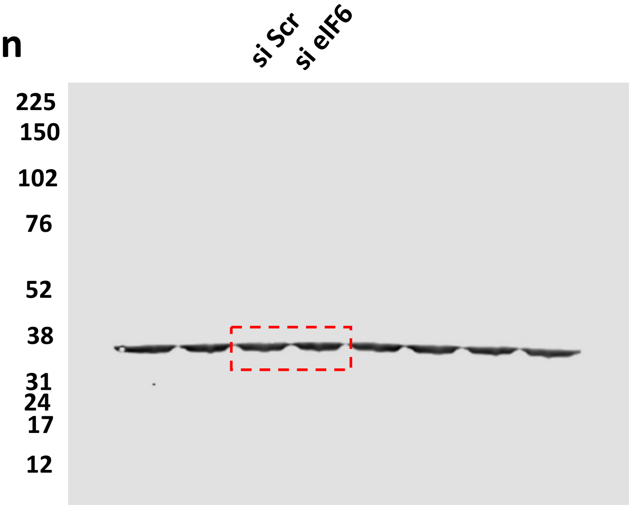

GAPDH

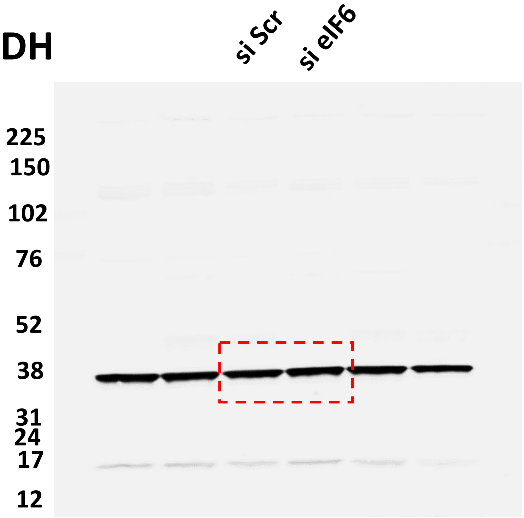

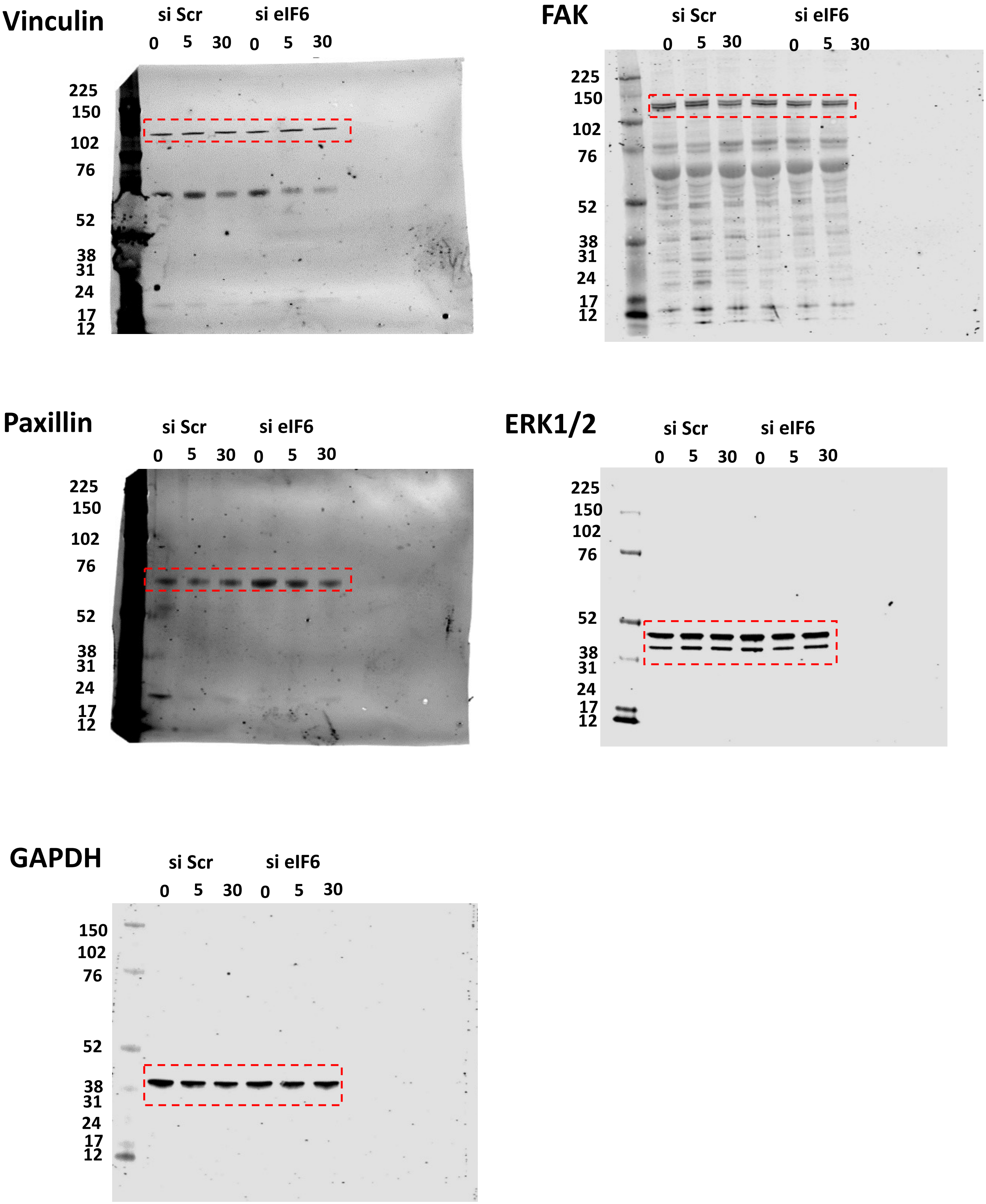
Vinculin
si Scr
si eIF6
0 5 30 0 5 30
225
150
102
76
52
38
31
24
17
12
FAK
si Scr
si eIF6
0 5 30 0 5 30
225
150
102
76
52
38
31
24
17
12
Paxillin
si Scr
si eIF6
0 5 30 0 5 30
225
150
102
76
52
38
31
24
17
12
ERK1/2
si Scr
si eIF6
0 5 30 0 5 30
225
150
102
76
52
38
31
24
17
12
GAPDH
si Scr
si eIF6
0 5 30 0 5 30
150
102
76
52
38
31
24
17
12
